# Replenished microglia partially rescue schizophrenia-related stress response

**DOI:** 10.3389/fncel.2023.1254923

**Published:** 2023-09-12

**Authors:** Ling Yan, Fang-Ling Xuan, Song Chen, Mengzhuang Gou, Wenjin Chen, Yanli Li, Zhiren Wang, Leilei Wang, Ting Xie, Fengmei Fan, Alexander Zharkovsky, Yunlong Tan, Li Tian

**Affiliations:** ^1^Institute of Biomedicine and Translational Medicine, Faculty of Medicine, University of Tartu, Tartu, Estonia; ^2^Psychiatry Research Centre, Beijing Huilongguan Hospital, Peking University Health Science Center, Beijing, China

**Keywords:** microglial replenishment, synaptic pruning, prefrontal cortex, hippocampus, chronic unpredictable stress, schizophrenia

## Abstract

**Background:**

Microglia play an important role in the maintenance of brain and behavioral homeostasis. The protective effect of microglial replenishment was reported in neurological diseases, but whether microglial therapy would benefit psychiatric disorders such as schizophrenia has been unclear. As schizophrenia is a stress-vulnerable disorder and psychosocial stress promotes inflammation and microglial activation, we aim to understand how microglial replenishment works in stress-associated schizophrenia.

**Methods:**

We used a CSF1R-mediated pharmacological approach to study repopulated microglia (repMg) in a cohort of mice (*n =* 10/group) undergoing chronic unpredictable stress (CUS). We further studied a cohort of first-episode schizophrenia (FES, *n =* 74) patients who had higher perceived stress scores (PSS) than healthy controls (HCs, *n =* 68).

**Results:**

Reborn microglia attenuated CUS-induced learned hopelessness and social withdrawal but not anxiety in mice. Compared to control, CUS- or repMg-induced differentially expressed genes (DEGs) in the prefrontal cortex regulated nervous system development and axonal guidance. CUS also caused microglial hyper-ramification and increased engulfment of synaptophysin and vesicular glutamate transporter-2 by microglia and astrocytes, which were recovered in CUS + repMg (all *p* < 0.05). Moreover, FES patients had smaller hippocampal fimbria than HCs (*p* < 1e-7), which were negatively associated with PSS (*r* = −0.397, *p* = 0.003). Blood DEGs involved in immune system development were also associated with PSS and the right fimbria more prominently in FES patients than HCs (Zr, *p* < 0.0001). The *KCNQ1* was a partial mediator between PSS and fimbria size (*β* = −0.442, 95% CI: −1.326 ~ −0.087).

**Conclusion:**

Microglial replenishment may potentially benefit psychiatric disorders such as schizophrenia.

## Introduction

Elimination of microglia by an inhibitor of colony-stimulating factor-1 receptor (CSF1Ri), such as PLX3397 or PLX5562, has been extensively studied. CSF1Ri leads to >95% elimination of microglia within 7–21 days, followed by rapid microglial replenishment upon drug withdrawal (Green et al., 2020). Repopulated microglia can improve behavioral, cognitive, inflammatory, and synaptic outcomes in several animal models of neurological diseases. Thus, microglial replenishment is regarded as a beneficial therapeutic approach for these diseases (Han et al., 2019; Green et al., 2020). For psychiatric disorders, the role of microglia ablation and replenishment is more complicated (Elmore et al., 2015; Torres et al., 2016; Rosin et al., 2018; Lehmann et al., 2019; Weber et al., 2019; Wang et al., 2020; Zhang et al., 2020; Ikezu et al., 2021). Some studies have demonstrated that microglial elimination does not affect or alleviate anxiety, social withdrawal, and memory deficit in animal stress models (Elmore et al., 2015; Lehmann et al., 2019; Weber et al., 2019; Wang et al., 2020; Ikezu et al., 2021). Nevertheless, others have observed the harmful effect of microglial elimination on psychiatric-like behaviors (Torres et al., 2016; Rosin et al., 2018; Zhang et al., 2020), highlighting the beneficial function of microglia. The role of microglia replenishment in psychiatric conditions remains similarly obscure in the available limited studies (Elmore et al., 2015; Rosin et al., 2018; Lehmann et al., 2019; Weber et al., 2019). Similarly, in schizophrenia, microglial activation in such patients has been implicated in some but not all studies (Sellgren et al., 2019; Conen et al., 2021; Rahimian et al., 2021; Gober et al., 2022).

The abovementioned miscellaneous observations may be at least partly due to mingled pathophysiological abnormalities caused by psychosocial stress in psychiatric disorders. Chronic psychosocial stressors have been profoundly demonstrated to cause the onset and development of psychiatric disorders as well as induce microglial activation and neuroinflammation (Koo and Wohleb, 2021). For schizophrenia, numerous studies have demonstrated that exposure to a traumatic life event(s) or psychological stress during the perinatal or adolescent period triggers or exacerbates symptoms (Pruessner et al., 2011; Holtzman et al., 2013; Gomes and Grace, 2017; Studerus et al., 2021). Schizophrenia patients commonly have dystrophies in the limbic system, which compromise their stress-coping and social-cognitive capabilities (White et al., 2008; Franklin et al., 2012). Recently, we reported that stress maladaptation, as reflected by allostatic load, is a composite index including blood immune components and was associated with cortical thinning and cognitive deficits in first-episode schizophrenia (FES) patients (Zhou Y. et al., 2021). We also found that stress maladaptation, as well as cortical and cognitive deficits, were linked to the enlargement of the choroid plexus – an important cerebral neuroimmune portal – in schizophrenia patients (Zhou et al., 2020; Huang et al., 2022). This large body of evidence found by others and us motivated us to understand microglia-related innate immune functions and their potential association with schizophrenia more deeply through the lens of chronic stress using animal models.

Chronic stress is well acknowledged to induce psychosis-related behaviors in rodent stress models, which have also been used to address microglial dysfunction in relation to psychiatric disorders, including schizophrenia (Koo and Wohleb, 2021). Specifically, microglia exposed to chronic unpredictable stress (CUS) showed increased phagocytosis of neuronal elements, leading to reduced dendritic spine density of pyramidal neurons (Wohleb et al., 2018). Microglial elimination protected mice from anxiety and depression-like behaviors caused by CUS or social defeat (Elmore et al., 2018; Lehmann et al., 2019; Wang et al., 2020; Ikezu et al., 2021). However, the precise role of microglia after replenishment in these paradigms has been obscure.

Thus, a better understanding of microglia–neuron interaction in schizophrenia in the context of stress association is required for the development of appropriate microglia-based therapy. The present study aims to characterize how repopulated microglia regulate animal psychiatric-like behaviors and the underlying mechanisms using a mouse model of CUS combined with CSF1Ri. Our underlying hypothesis is that reborn microglia manifest reprogrammed functions that may reflect myeloid alterations in stress-related schizophrenia. We thereby performed a series of animal experiments to fine-map microglial properties in the prefrontal cortex (PFC) and hippocampus. We further studied those FES patients who showed heightened stress perception and innate immune activation in comparison to healthy subjects (Chen et al., 2022; Xuan et al., 2022).

## Materials and methods

### Animals

Wild-type C57BL/6NTac male mice (3-months-old, Taconic) were bred in a laboratory animal facility at the Institute of Biomedicine and Translational Medicine, University of Tartu. Mice from different litters were housed in 1264C Euro standard type II cages (Tecniplast S.p.A.) measuring 268 × 215 × 141 mm. Cages containing aspen chips and wool for bedding and nesting were replaced once a week. Each cage contained 9–10 animals based on allocation after weaning. Mice were kept under standard conditions with unlimited access to food and water on a 12/12-h light/dark cycle (light on from 7 a.m. to 7 p.m.). All animal procedures in this study were performed in accordance with the European Communities Directive with license No. 171 issued by the Estonian National Board of Animal Experiments.

### CUS and PLX3397 treatment procedures

**Figure 1:**
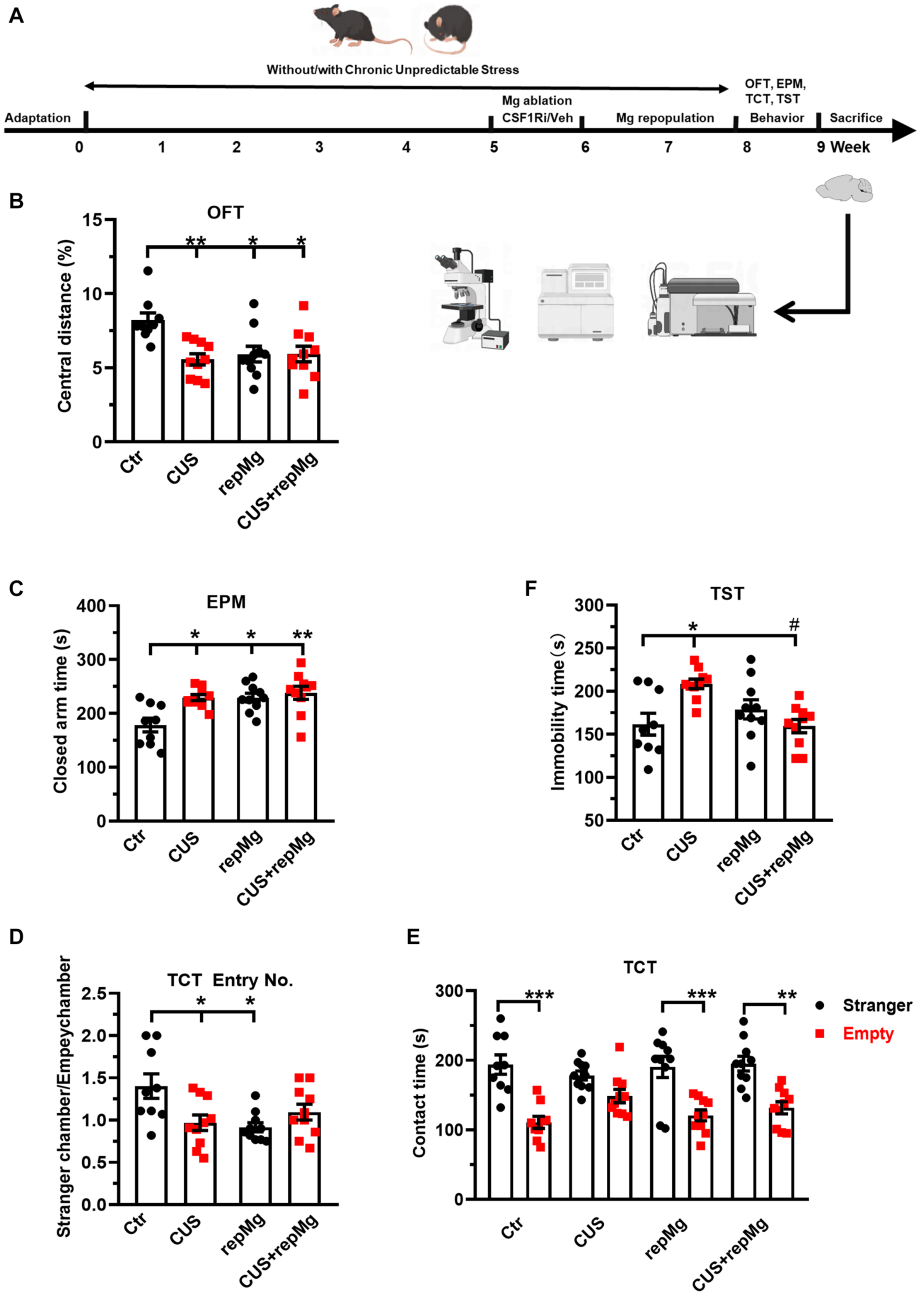
RepMg rescued CUS-induced learned helplessness and social deficit but not anxiety. **(A)** Schematic diagram of experimental design by Figdraw. Mice were separated into four groups (Ctr, CUS, repMg, and CUS + repMg; *n* = 9/10/10/10 pergroup) and underwent CUS and/or CSF1Ri (PLX3397) treatment. Afterward, behavioral tests were conducted, followed by flow cytometry and IHC. **(B)** Treated mice showed decreased central versus total distance (%) than Ctr mice in OFT. **(C)** In the EPM test, time spent in closed arms was significantly increased in CUS, repMg, and CUS + repMg. **(D,E)** In TCT, ratios of frequencies of entry into a stranger mouse chamber versus an empty container chamber were declined by CUS or repMg treatment but not CUS + repMg. **(D)** Moreover, except for CUS, the other groups showed similarly longer stay with the stranger mice than with the empty container **(E)**. **(F)** In TST, CUS induced elongated immobility time compared to Ctr, which was reduced by CUS + repMg. Mean ± SEM; * or # *p* < 0.05, ** *p* < 0.01 (* compared to Ctr; # compared to CUS). Two-way ANOVA with Tukey’s corrections.

Mice were randomly assigned into four groups: Control (Ctr) (*n* = 9), CUS (*n* = 10), microglial repopulation (repMg) (*n* = 10), and CUS + repMg (*n* = 10). After a week of transfer adaptation, the mice were subject to CUS/Ctr and CSF1Ri/Veh treatments (Figure 1A and Supplementary Figure S2B). CUS procedures, as previously described (Cangalaya et al., 2020; Chen et al., 2022; Xuan et al., 2022), were followed. Briefly, mice were exposed to a variable sequence of seven mild and unpredictable stressors once per day (d) for eight consecutive weeks, including food and water deprivation overnight, rat odor and isolation overnight, restraint in a 50-ml tube for 2 h, wet bedding and tilted cage, stroboscopic illumination overnight, flipped light/dark exposure, and swimming at 18°C for 10 min. All stressors were randomly scheduled and changed daily to sustain an unpredictable procedure.

PLX3397 (HY-16749/CS-4256, MedChemExpress) was dissolved at 200 mg/mL in DMSO (D8418, Sigma-Aldrich) stock solution, and an aliquot was freshly diluted with corn oil (#8267, Sigma-Aldrich) by 1: 6.5 before using. Drug-treated mice were individually fed with Veh (100 μL 15% DMSO/85% corn oil+0.5 g Nutella/mouse/day) or PLX3397 (120 mg/kg body weight, e.g., 3 mg/mouse in Veh) (Cangalaya et al., 2020) for voluntary ingestion for 7 consecutive days after 5 weeks of CUS, followed by a 2-week recovery period.

### Open field test (OFT)

Mice were habituated to ~250 lux room light for 1 h. Each mouse was measured for distance and time traveled in different zones of a digitalized box (44.8 × 44.8 × 45 cm) via software (Technical & Scientific Equipment GmbH, Germany) for 30 min. The floor of the box was cleaned with 70% ethanol and dried thoroughly after each mouse.

### Elevated plus maze (EPM)

The elevated plus maze consisted of open and closed arms (30 × 5 cm each) intersected at a central 5 × 5-cm square platform elevated to a height of 80 cm. Mice were habituated to ~40 lux room light for 1 h. Each mouse was placed on the central platform facing the open arm and recorded for time spent on open or closed arms using EthoVision XT software (Nodules, United States) for 5 min. The arms were cleaned with 70% ethanol and dried thoroughly after each mouse.

### Three-chamber test (TCT)

Mice were habituated to ~40 lux room light for 1 h. A rectangular three-chamber box made from clear Plexiglas was divided into three identical sections, each side accommodating a lid-covered and wire-structured cup-like container large enough to enclose a single mouse, allowing free exchange of air but not direct physical contact between mice on both sides. A test mouse was first habituated in the central chamber for 5 min and then introduced to a stranger mouse located in a container and left to freely explore the three chambers for the next 10 min. All stranger mice were at the same age as test mice and habituated to the apparatus in advance. The box and wire containers were cleaned with 70% ethanol and dried thoroughly after each test. Social preference was defined as time spent with the stranger mouse, and social contacts were counted when the head and front paws of a test mouse were within 3 cm vicinity of the container wall as recorded by a camera (Nodules, United States).

### Tail suspension test (TST)

Mice were habituated to ~40 lux room light for 1 h. An animal was hung on a wooden bar by the tip of the tail using an adhesive tape and recorded with a camera for 5 min. A complete lack of movement or small movements of forefeet and body swinging was counted as immobility, and time (s) of immobility was measured.

### RNA sequencing (RNA-seq) data analysis

Mice were euthanized with CO_2,_ and the PFC were dissected and immediately stored at −80°C. Human peripheral blood was collected in PAXgene^™^ blood RNA tubes. Total RNAs from human blood (Applied Biosystems, United States) and mouse PFC (Molecular Research Center, United States) were extracted, quantified, assessed for purity using a NanoDrop spectrophotometer (Thermo Fisher Scientific, United States) and immediately sent to the Beijing Genomics Institution (BGI) for messenger RNA-seq (after globin mRNA removal and quality control) on the BGIseq-500 platform. The quality of the RNA samples (RIN/RQN ≥ 7.0, 28S/18S ≥ 1.0) was confirmed by BGI. Clean data of at least 20 M clean reads per sample were collected.

RNA-seq data and gene expression analysis were done on the Galaxy and NetworkAnalyst platforms using DESEQ2 (Zhou et al., 2019). Data with variance percentile rank of <15% and counts of <4 were filtered out. Counts per million reads were transformed, normalized, and calculated to Log2 fold changes (Log2FC) for differentially expressed genes (DEGs). DEGs with Benjamini-Hochberg’s false discovery rate (FDR) of <0.05 were subjected to functional clustering analysis in DAVID.^1^

### Immunohistochemistry (IHC)

C57BL/6Ntac mice were anesthetized with an intraperitoneal injection of end-dose of ketamine or xylazine mixture and transcardially perfused with PBS and 4% paraformaldehyde (PFA). Dissected brains were post-fixed in the 4% PFA at 4°C for 1 day, dehydrated by 30% sucrose, and stored at −80°C. The brains were taken to −20°C for 1 day before cryosectioning.

PFC coronal cryosections in 40 μm-thickness were washed in PBS and incubated in 0.5% triton-x100. After PBS washing, slices were incubated with primary antibodies, including rabbit anti-IBA1 (#SKL6615, Wako, United States, diluted 1:500) and mouse anti-synaptophysin (SYP, sc-17,750, Santa Cruz, United States, diluted 1:250) in blocking buffer (10% goat serum+1%BSA+ 0.3 M Glycine) overnight at 4°C. It was followed by PBS washing and incubation with the secondary antibodies, including goat anti-rabbit IgG H&L-Alexa568 (#ab175471, Abcam, United States), goat anti-mouse IgG H&L-PECy7 (#D2110, Sant Cruz, United States, diluted 1:500), and 0.1 μg/mL DAPI (#ACRO202710100, VWR, United States) for 2 h at room temperature. After PBS washing, slices were mounted onto glass slides with Fluoromount^™^ Aqueous Mounting Medium (#F4680-25ML, Sigma-Aldrich, United States).

Images of 512 × 512 pixels were taken by an FV1200MPE laser scanning microscope at 60× magnification (Olympus, United States) with a scanning velocity of 12.5 pixel/μm and Z-stacks (step: 0.5 μm, depth: 20 μm) to analyze the morphology of IBA1^+^ cells and puncta of SYP. The SNT package in ImageJ was used to reconstruct 3D cell morphology, and “Sholl analysis” was used to acquire the ramification index, while the other parameters were acquired through “quick measurements” of SNT, including total branch length and the number of branches. SYP^+^ puncta in the microglial region of interest defined via cell skeleton were obtained by “analyze particles,” and SYP density was calculated as SYP^+^ area (μm^2^) per image area (3.03 × 103 μm^2^) or per microglial area. 3D images segregating IBA1^+^ microglia, nuclei, and SYP^+^ puncta were obtained with “surface” and “spots” functions with videos created by “animation” in Bitplane software (Imaris).

### Flow cytometry

Mouse hippocampi were dissected after CO_2_ euthanization and gently homogenized through 70 μm cell strainers (#352350, BD Bioscience, United States) in ice-cold PBS with 1% FBS. Homogenates were washed and centrifuged at 500 g for 5 min. Isolated cells were blocked with 10% rat serum in ice-cold PBS for 1 h. Brain cells were stained with 0.5 μL anti-mouse VGLUT2-Alexa488 (#MAB5504A4, Millipore, United States), CD11b-BV421 (#101251, BioLegend, United States), CD45-BV650 (#103151, BioLegend, United States), Glast-APC (#130–123-555, Miltenyi, Germany), and O4-PE (#130–117-357, Miltenyi, Germany) in PBS with 1% FBS on ice for 1 h. Corresponding isotype control antibodies (all BioLegend, United States) included rat IgG2a-Alexa488 (##400,525), IgG2b-BV421 (#400639), IgG2b-BV650 (#400651), IgG2b-APC (#400219), and IgM-PE (#401611). Cells were washed, resuspended in 500 mL PBS, and acquired with a Fortessa flow cytometer (BD Bioscience, United States). Data were analyzed by Kaluza v2.1 software (Beckman Coulter, United States). Astrocytes were defined as Glast^+^ cells, oligodendrocyte precursor cells (OPCs) as O4^+^ cells, and microglia as CD45^low^CD11b^hi^ cells. Cell populations were calculated as % among total brain cells or microglia as previously described (Piirainen et al., 2021; Chithanathan et al., 2022).

### Participants’ demographic and clinical measures

FES patients (*n* = 74) were recruited from the Beijing Huilongguan Hospital. Patients were diagnosed with schizophrenia according to the Structured Clinical Interview for DSM-IV (SCID) independently by two psychiatrists. The inclusion criteria were as follows: (Green et al., 2020) 18-54-year-old Han Chinese people; (Han et al., 2019) illness duration of ≤3 years (<1 year on average); and (Wang et al., 2020) un-medicated or < 2 weeks of anti-psychotic medication at the time of blood draw. HCs (*n* = 68) matched for age and sex were recruited from the local community. Candidates who did not meet the recruitment criteria were excluded. All participants provided written informed consent. The study was approved by the Institutional Ethical Committee of Beijing Huilongguan Hospital (No.2017–49).

Past traumatic experiences were evaluated by a childhood trauma questionnaire (CTQ) constituting retrospectively measured 29 items encompassing the following five factors: physical abuse, emotional abuse, sexual abuse, physical neglect, and emotional neglect. Stress levels were evaluated based on a perceived stress scale (PSS) questionnaire constituting 14 items measuring feelings and thoughts during the past month. Sub-scores and total scores (PANSS-P, -N, −G, −T) of the positive and negative syndrome scale were measured independently by two psychiatrists.

### Magnetic resonance imaging (MRI)

Brain structural MRI data were acquired using a Siemens Prisma 3.0 T MRI scanner with a 64-channel head coil. Foam pads were used to minimize head motions. Sagittal 3D magnetization-prepared rapid acquisition gradient echo (MPRAGE) was chosen for imaging, using ENIGMA protocol and FreeSurfer software, with repetition time (TR)/echo time (TE)/inversion time (TI) = 2530/2.98/1100 ms, flip angle (FA) = 7°, field of view (FOV) = 256 × 224 mm^2^, pixel/gap size = 1/0 mm, and matrix size = 256 × 224 bit. After scanning, two radiologists evaluated image quality, and if significant artifacts existed, images were recollected. Representative T1 MRI images at −16 mm from the bregma were obtained from “The Scalable Brain Atlas” (Bakker et al., 2015). Subregions of the hippocampus were drawn based on the Allen Human Brain Atlas.^2^

### Statistical analysis

Data distributions were examined through the Shapiro–Wilk test. For animal data, two-way ANOVA was used to examine the interaction between CUS and repMg and main effects, with Tukey’s or LSD correction for post-hoc pairwise comparisons. For human data, ANOVA or the Mann–Whitney U test was used for continuous variables and the chi-squared tests for categorical variables. ANCOVA was conducted for MRI data, and correlational analyzes were done using Spearman’s or Pearson’s partial method, with age, sex, and BMI as covariates using SPSS-v27.0 (IBM). Figures were prepared in GraphPad Prism v8.0.1 and online.^3^ Data were presented as mean ± SEM, and a *p*-value of <0.05 was considered statistically significant.

## Results

### Replenished microglia rescue CUS-triggered learned hopelessness and social deficit but not anxiety

To evaluate the effects of CUS and repMg, we performed a battery of mouse behavioral tests (Figure 1A). CUS induced anxiety, as shown by a decreased ratio of central versus total travel distance (*p* < 0.01, Figure 1B) in OFT and increased time in EPM closed arms (*p* < 0.05, Figure 1C). In RepMg and CUS + repMg, mice showed similar anxiogenic effects in OFT (both *p* < 0.05, Figure 1B) and EPM (*p* < 0.05/0.01, Figure 1C). CUS x repMg interactions were found in OFT (*F* (1, 35) = 7.545, *p* < 0.01) and EPM (*F* (1, 35) = 4.489, *p* < 0.05). Overall, it suggested that microglial replenishment itself caused anxiety and cannot ameliorate CUS-induced anxiety.

A CUS x repMg interactive effect on sociability was also found in TCT (*F* (1, 35) = 9.623, *p* < 0.01). CUS mice displayed less preferential entry into the social chamber and spent time there than in the empty chamber (Figures 1D,E). Mice in the repMg group also entered less into the social chamber (Figure 1D) but spent a longer time once there than in the empty chamber (*p* **<** 0.001, Figure 1E). However, mice of the CUS + repMg group showed normal sociability similar to Ctr mice (*p* < 0.01, Figures 1D,E), indicating that repMg can partially rescue CUS-triggered social deficit.

For depression-like behavior, CUS also interacted with repMg (*F* (1, 35) = 12.180, *p* < 0.01). Mice in CUS, but not repMg, showed longer immobility time than Ctr mice (*p* < 0.05, Figure 1F). Furthermore, CUS + repMg mice showed significantly less immobility time than CUS mice (*p* < 0.05, Figure 1F), demonstrating the ameliorative effect of repMg on CUS-induced learned helplessness.

### CUS and microglial replenishment affect brain developmental transcriptomics in the mouse PFC

**Figure 2:**
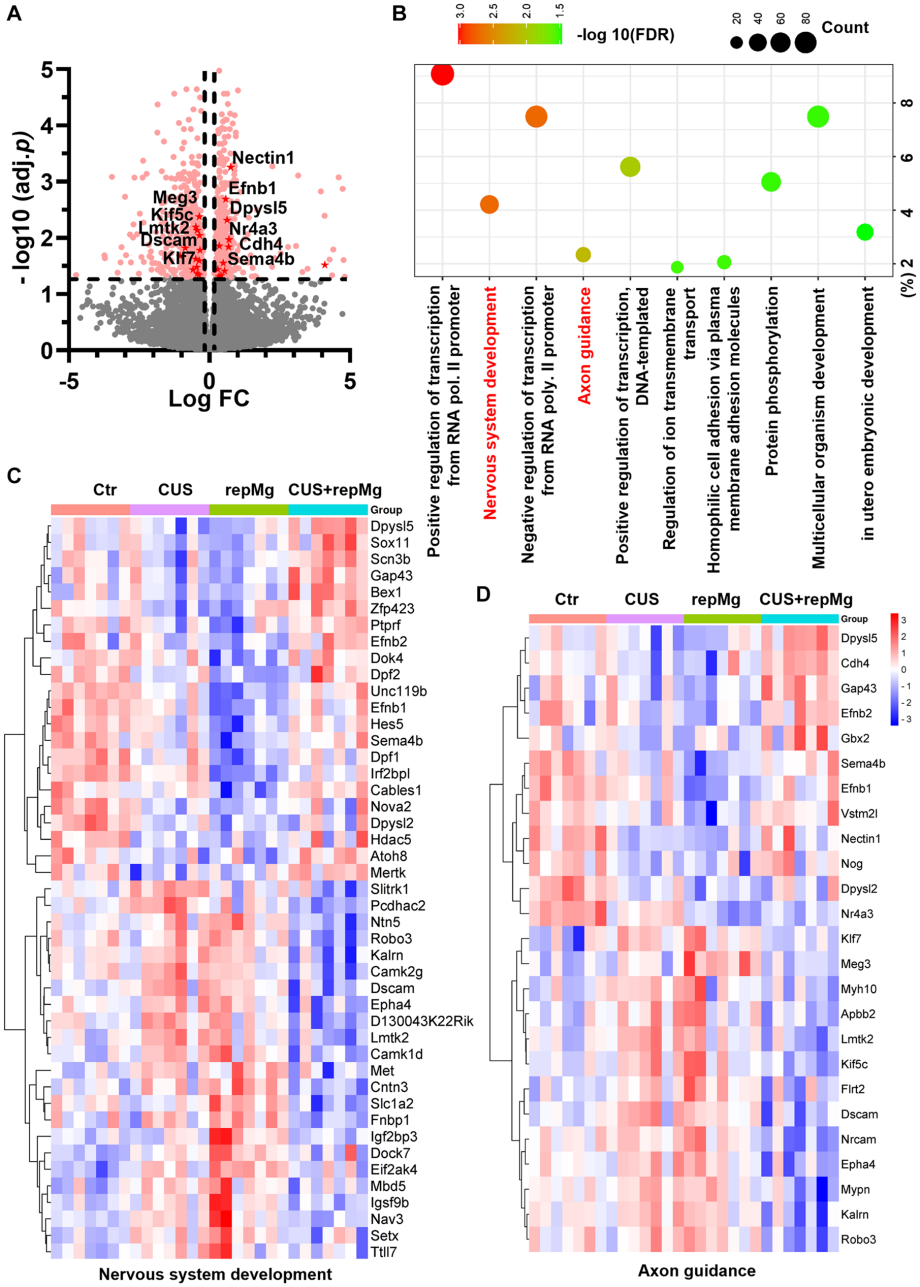
CUS and repMg changed DEGs involved in nervous system development and axonal guidance in the mouse PFC. **(A)** Volcano plot showing expressions of DEGs compared among the four groups (Ctr, CUS, repMg, and CUS + repMg; *n* = 7/group) after bulk RNA-seq. Pink dots indicate significant DEGs. Those involved in axon guidance and with values of −log10(FDR) > 1.3 are highlighted in red dots. **(B)** GO-BP enrichment analysis of the DEGs showing top 10 pathways based on -log10(FDR) clustering values and gene counts. **(C,D)** Heatmaps showing expressions of DEGs involved in nervous system development and axon guidance, which were changed in CUS and repMg compared to Ctr but partially recovered in CUS + repMg. Two-way ANOVA with FDR. See also Supplementary Tables S1–S3 and Supplementary Figure S1.

Among the four groups, 502 upregulated and 577 downregulated DEGs were identified, involving nervous system development and axon guidance among the top 10 GO-BP pathways (Figures 2A–D and Supplementary Table S1). Notably, expressions of the DEGs in these two pathways were generally recovered in CUS + repMg (Figures 2C,D). Furthermore, compared to Ctr, DEGs particularly affected by repMg were involved in the rhythmic process and regulation of synaptic plasticity, with most synaptic DEGs down-regulated (Supplementary Figure S1 and Supplementary Table S2), whereas those affected by CUS were a small group of angiogenic DEGs (Supplementary Table S3). These results reveal that CUS and repMg both affect brain structural remodeling, but repMg can partially restore brain developmental processes altered by CUS.

### Replenished microglia correct CUS-triggered presynaptic endocytosis by glial cells in the PFC and hippocampus

**Figure 3:**
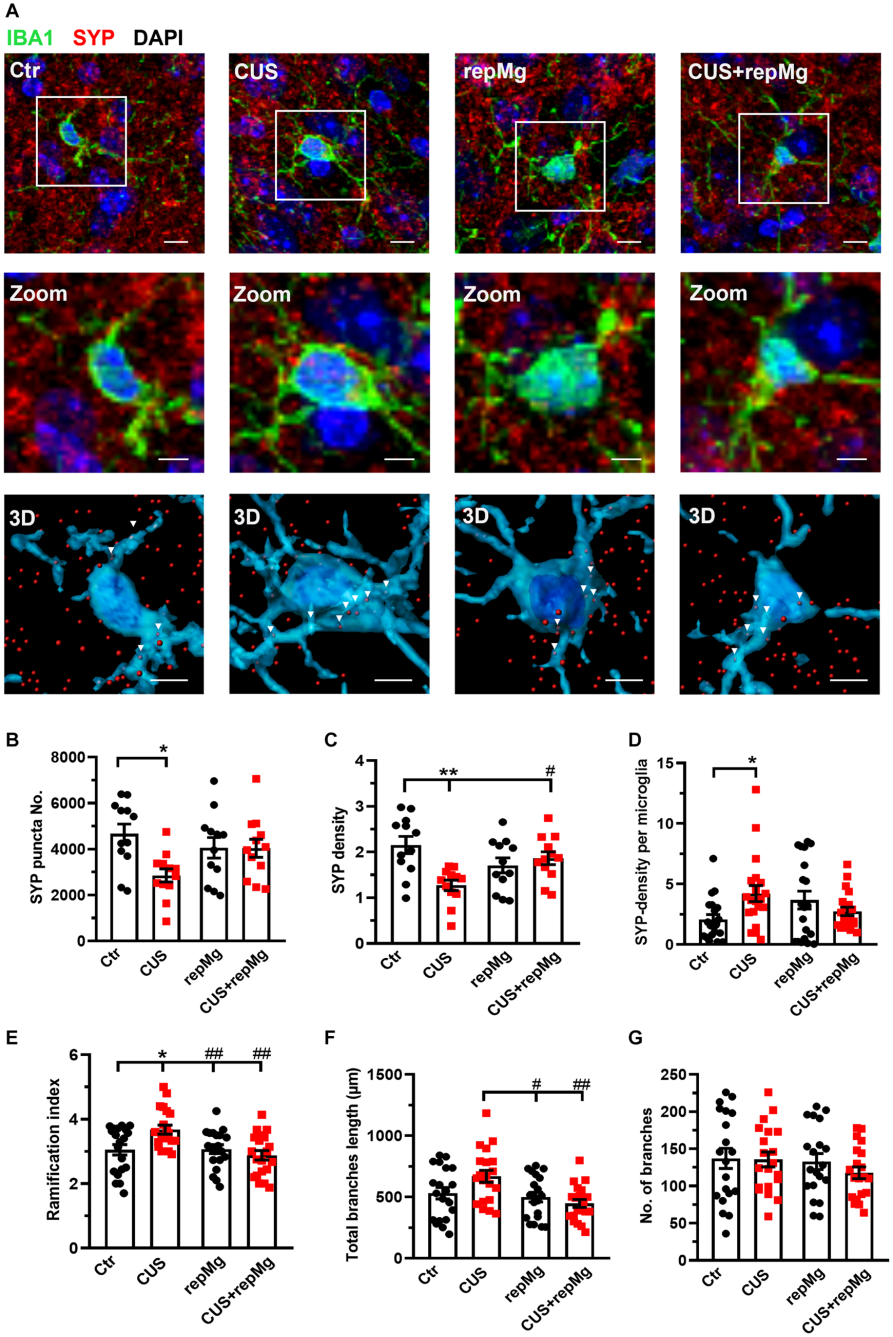
RepMg rescued CUS-triggered loss of SYP and microglial over-ramification in the mouse PFC. **(A)** Representative images of microglia (IBA1, green), synaptophysin (SYP, red), and nucleus (DAPI, blue), with zoomed and 3D images showing SYP puncta in microglia. Arrowheads indicate SYP puncta engulfed by microglia. Scale bar = 1 μm. **(B)** CUS decreased SYP puncta compared to Ctr. **(C)** CUS reduced SYP density, which was recovered in CUS + repMg. **(D)** CUS enhanced SYP density in microglia. **(E)** CUS enhanced microglial ramification compared to Ctr, which was dampened in repMg and CUS + repMg. **(F)** Microglial total branch length was shorter in repMg and CUS + repMg than in CUS. **(G)** Number of microglial branches did not change. Mean ± SEM; * or # *p* < 0.05, ** or ## *p* < 0.01 (* compared to Ctr; # compared to CUS). Two-way ANOVA with Tukey’s corrections.

To verify the synaptic remodeling function of replenished microglia, we then studied the mouse PFC and hippocampal regions. We first co-stained mouse PFC sections by IHC for SYP, a presynaptic marker, and IBA1 (Figure 3A). CUS x repMg interactions were found on SYP punctal number (*F* (1, 44) = 5.229, *p* < 0.05), SYP density (*F* (1, 44) = 11.480, *p* < 0.01), and SYP-density per microglia (*F* (1,76) = 7.469, *p* < 0.01). CUS-induced loss of SYP puncta (*p* < 0.05, Figure 3B) and SYP density (*p* < 0.01, Figure 3C) compared to Ctr, and repMg rescued CUS-triggered SYP decrease (*p* < 0.05, Figure 3C). Importantly, SYP density inside microglia was higher in CUS than Ctr (*p* < 0.05, Figure 3D).

CUS and repMg also interacted on microglial ramification index (*F* (1,76) = 7.963, *p* < 0.01) and total branch length (*F* (1,76) = 5.089, *p* < 0.05). Compared to Ctr, CUS-conditioned microglia had a larger ramification index (*p* < 0.05, Figure 3E) and longer total branch length (*p* < 0.01, Figure 3F). In contrast, repMg corrected CUS triggered increases in these parameters (both *p* < 0.01, Figures 3E,F). No differences were found in the number of branches among the groups (Figure 3G).

**Figure 4:**
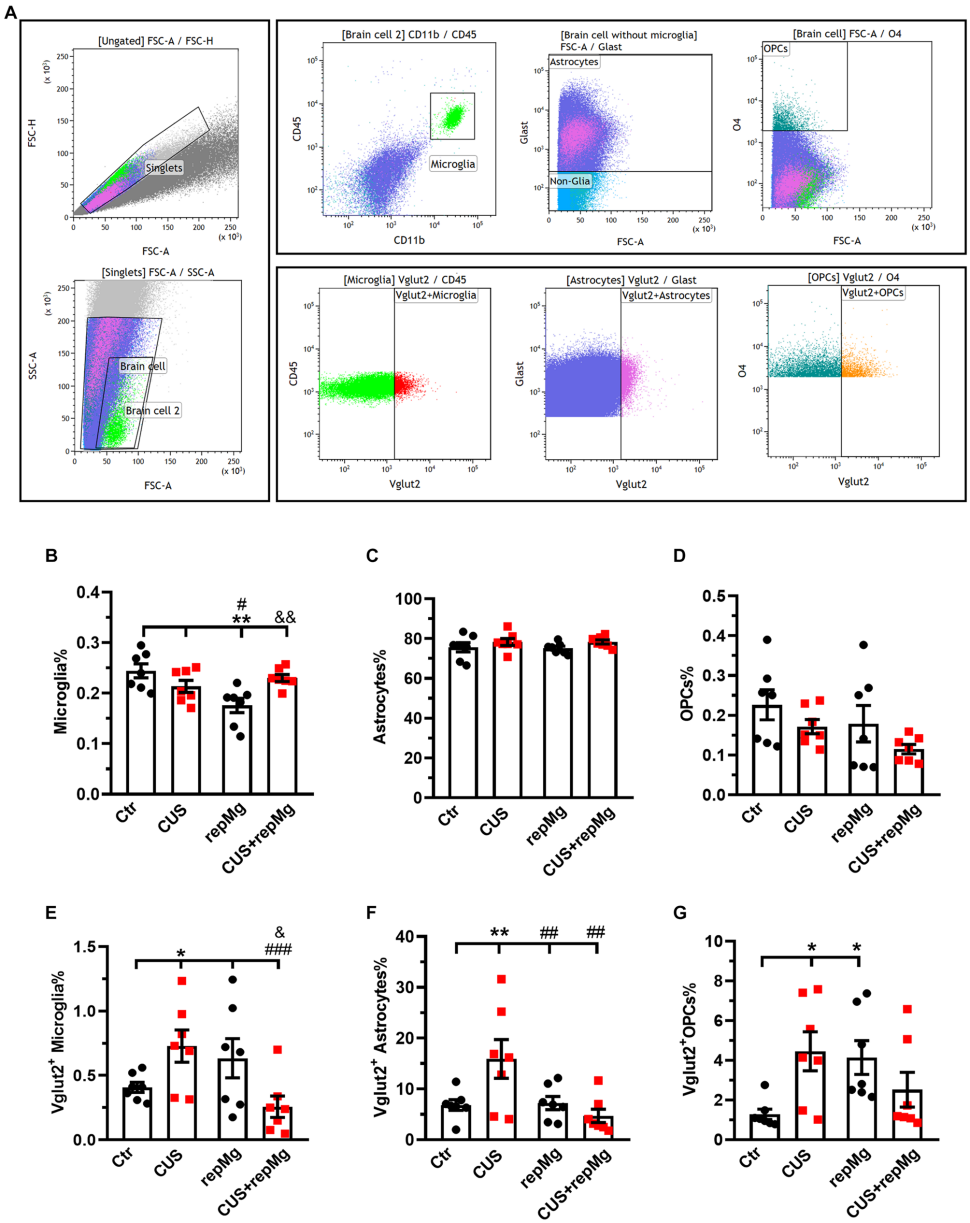
CUS and repMg enhanced endocytosis of VGLUT2^+^ by hippocampal microglia, astrocytes, or OPCs. **(A)** Dot plots represent the gating strategy of flow cytometric analysis. **(B)** Compared to Ctr and CUS, microglia in repMg were still less abundant but recovered better in CUS + repMg. **(C,D)** Abundancies of astrocytes and OPCs did not change. **(E–G)** CUS induced universal VGLUT2 engulfment by all three glial subtypes compared to Ctr, which was ameliorated in repopulated microglia **(E)** and astrocytes **(F)** in CUS + repMg. Furthermore, repMg had more abundant VGLUT2^+^ microglia than CUS + repMg (E), VGLUT2^+^ astrocytes than CUS group **(F)**, and VGLUT2^+^ OPCs than Ctr **(G)**. Mean ± SEM; * or # or & *p* < 0.05, ** or ## or && *p* < 0.01, ### *p* < 0.001 (* compared to Ctr; # compared to CUS; & compared to repMg). Two-way ANOVA with LSD corrections. See also Supplementary Figure S2.

We also quantified glial cells by flow cytometry with hippocampal tissues, another important region for stress regulation (Figure 4A and Supplementary Figure S2A). CUS and repMg interacted on microglia% (*F* (1,24) = 10.680, *p* < 0.01). However, CUS did not significantly affect microglia% (Figure 4B). Although microglial abundancy did not fully recover in repMg as compared to Ctr (*p* < 0.01, Figure 4B), the recovery was better in CUS + repMg compared to repMg alone (*p* < 0.01, Figure 4B). No changes in other glial populations were found (Figures 4C,D), demonstrating the microglia-specific effect of CSF1Ri.

We further quantified the glial engulfment of VGLUT2, a glutamatergic presynaptic marker. CUS x repMg interactions existed on VGLUT2^+^ microglia (*F* (1, 24) = 10.250, *p* < 0.01), VGLUT2^+^ astrocytes (*F* (1, 24) = 7.024, *p* < 0.05), and VGLUT2^+^ OPCs (*F* (1, 24) = 9.100, *p* < 0.01). Compared to Ctr, CUS elevated VGLUT2 in microglia (*p* < 0.05, Figure 4E), astrocytes (*p* < 0.01, Figure 4F), and OPCs (*p* < 0.05, Figure 4G).

Compared to Ctr, replenished microglia were not over-pruning but led to elevated VGLUT2 engulfment by OPCs (*p* < 0.05, Figure 4G). Notably, repMg normalized VGLUT2^+^ engulfment by stressed microglia (*p* < 0.001, Figure 4E) and astrocytes (*p* < 0.01, Figure 4F), thereby supporting the RNA-seq and IHC results and strengthening the idea that repMg can partially rescue CUS-induced synaptic deficits.

These data overall suggest that CUS-conditioned microglia are over-ramified and more phagocytic on presynaptic components, which can be corrected by repMg.

### Hippocampal fimbria are smaller in FES patients and negatively correlated with stress perception and psychosis

We previously found that some FES patients had higher stress sensitivity (Xuan et al., 2022) and reduced amounts of nonclassical monocytes along with altered signature gene expressions (Chen et al., 2022) compared to HCs, implying their deficits in myeloid reprogramming. To relate the implications of repMg to a clinical setting, we explored a cohort from these FES patients and HCs.

**Figure 5:**
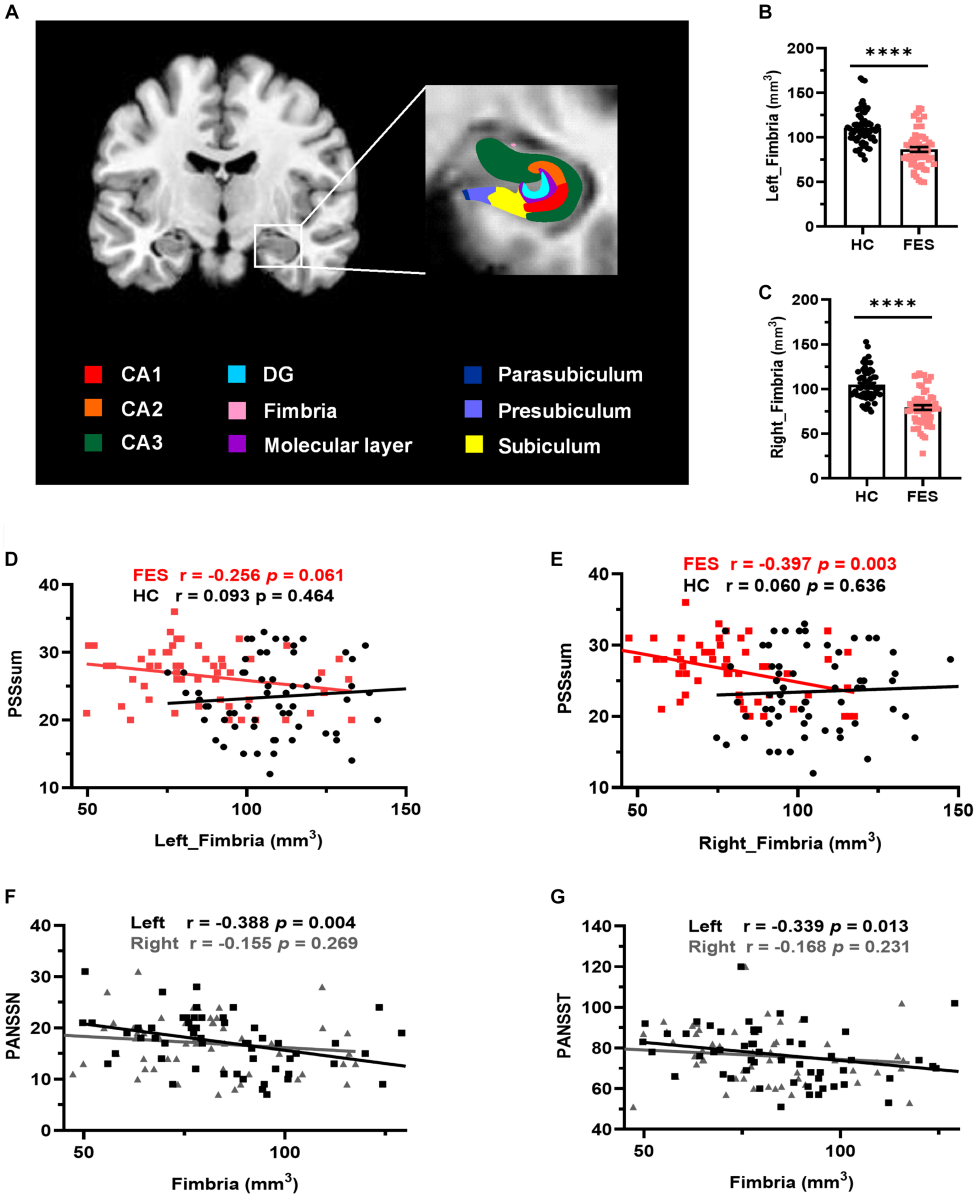
Hippocampal fimbria was smaller in FES patients and negatively correlated with PSS and PANSS scores. **(A)** A representative T1 MRI image with colored hippocampal subregions. **(B,C)** The bilateral fimbria was significantly reduced in FES patients (*n* = 59) compared to HCs (*n* = 64). Data are plotted as mean ± SEM; **** *p* < 0.0001. ANCOVA with age, sex, and BMI as covariates. **(D,E)** Volumes of the right fimbria were negatively correlated with PSS scores in FES patients but not HCs (Spearman’s). **(F,G)** Volumes of the left fimbria were negatively correlated with both PANSSN and PANSST scores (Pearson’s partial correlation controlled for age, sex, and BMI). See also Supplementary Tables S4–S6.

No differences in age, sex, and education years between the two groups were found (Table 1). However, FES patients had lower BMI (*p* < 0.001) as well as higher CTQ (*p* = 0.002, Table 1) and PSS (*p* = 0.001, Table 1) scores than HCs. Interestingly, MRI data showed alterations of hippocampal subregions (Figure 5A and Table 2), but not PFC structures (data not shown), in FES patients, with significantly smaller hippocampal fimbria (left: *p* = 7.213e-8, right: *p* = 1.041e-9, Figure 5B) but larger tails (left: *p* < 0.05, right: *p* < 0.0001), left presubiculum (*p* < 0.01), and right fissure (*p* < 0.05).

**Table 1:** Demographic characteristics of FES patients and HCs.

Demographics	FES (*n* = 74)	HC (*n* = 68)	*F* or 𝜒^2^	*p*
Sex (M/F)	44/30	31/37	2.736	0.098
Age (years)	31.30 (1.20)	33.77 (1.27)	2915.500	0.102
Education (years)	12.67 (0.45)	13.37 (0.31)	2847.000	0.167
BMI (kg/m^2^)	21.48 (0.400)	23.38 (0.39)	3382.500	**2.892E-5**
CTQsum	86.84 (4.59)	66.00 (3.62)	1336.500	**0.002**
PSSsum	26.93 (0.50)	23.19 (0.79)	1554.000	**0.001**
Age of illness onset (years)	29.90 (1.09)			
Illness duration (months)	14.89 (2.30)			
PANSSP	21.68 (0.59)			
PANSSN	17.72 (0.73)			
PANSSG	37.72 (0.97)			
PANSST	77.10 (1.64)			

BMI, body mass index; CTQsum, childhood trauma scale summation; PSSsum, perceived stress scale summation; PANSS, positive and negative symptom scale; PANSSP, the positive scale of PANSS; PANSSN, the negative scale of PANSS; PANSSG, general psychopathology scores of PANSS; PANSST, total scores of PANSS.

Bold values mean that *p*-values were significant.

**Table 2:** Hippocampal volumes (mm^3^) in FES patients and HCs.

Cortical regions	FES (*n* = 56)	HC (*n* = 64)	*F*	*p*
HP	8134.16 ± 85.18	8155.13 ± 90.73	0.168	0.683
Left_Whole HP	3443.81 ± 32.83	3438.20 ± 37.16	0.516	0.474
Right_Whole HP	3514.50 ± 36.04	3530.10 ± 34.99	0.130	0.719
Left_CA1	611.59 ± 7.86	610.02 ± 7.90	0.427	0.515
Left_CA3	185.54 ± 2.87	186.60 ± 3.23	0.037	0.849
Left_CA4	245.40 ± 2.95	250.59 ± 3.21	0.765	0.384
Left_DG	288.00 ± 3.34	294.15 ± 3.77	0.826	0.365
Left_Fimbria	86.62 ± 2.69	110.68 ± 2.43	33.152	**7.213E-8**
Left_Fissure	151.17 ± 2.80	146.53 ± 2.52	2.133	0.147
Left_HATA	55.01 ± 1.01	57.29 ± 1.05	1.089	0.299
Left_Molecular layer	563.52 ± 6.14	564.39 ± 6.24	0.204	0.652
Left_Parasubiculum	63.43 ± 1.71	61.50 ± 1.54	1.423	0.235
Left_Presubiculum	338.81 ± 5.75	320.17 ± 4.50	8.068	**0.005**
Left_Subiculum	447.56 ± 4.75	453.87 ± 5.55	0.025	0.875
Left_Tail	558.32 ± 8.80	528.93 ± 7.99	6.317	**0.013**
Right_CA1	628.94 ± 8.68	645.05 ± 7.34	0.755	0.387
Right_CA3	201.32 ± 3.45	201.26 ± 3.70	0.031	0.860
Right_CA4	253.29 ± 3.09	256.56 ± 3.23	0.152	0.697
Right_DG	296.09 ± 3.57	301.50 ± 3.67	0.438	0.509
Right_Fimbria	79.27 ± 2.58	104.78 ± 2.14	44.204	**1.041E-9**
Right_Fissure	155.84 ± 2.99	147.90 ± 2.55	6.664	**0.011**
Right_HATA	55.92 ± 1.00	58.31 ± 0.89	1.192	0.277
Right_Molecular layer	575.82 ± 6.84	585.05 ± 5.94	0.275	0.601
Right_Parasubiculum	57.46 ± 1.23	57.20 ± 1.47	0.080	0.777
Right_Presubiculum	319.92 ± 5.48	308.15 ± 3.90	3.811	0.053
Right_Subiculum	449.87 ± 5.71	461.16 ± 4.50	0.370	0.544
Right_Tail	596.61 ± 8.98	551.08 ± 7.83	16.520	**8.800E-5**

ANCOVA with age, sex, and BMI as covariates; DG, dentate gyrus; HP, hippocampus; HATA: hippocampal amygdala transition area.

Bold values mean that *p*-values were significant.

Volumes of the right hippocampal fimbria were negatively correlated with PSS scores in FES patients (*r* = −0.397, *p* = 0.003, Figure 5E and Supplementary Table S4) but not in HCs. While no correlations with PSS scores were found for the left hippocampal fimbria in both groups (Figure 5D), they were negatively associated with PANSS scores (PANSSN: *r* = −0.388, *p* = 0.004; PANSST: *r* = −0.339, *p* = 0.013; Figures 5F,G and Supplementary Table S5). No correlations were found between CTQ scores and hippocampal volumes (Supplementary Table S6).

### Blood immune DEGs are correlated negatively with PSS and positively with the right hippocampal fimbria in FES patients but not HCs

**Figure 6:**
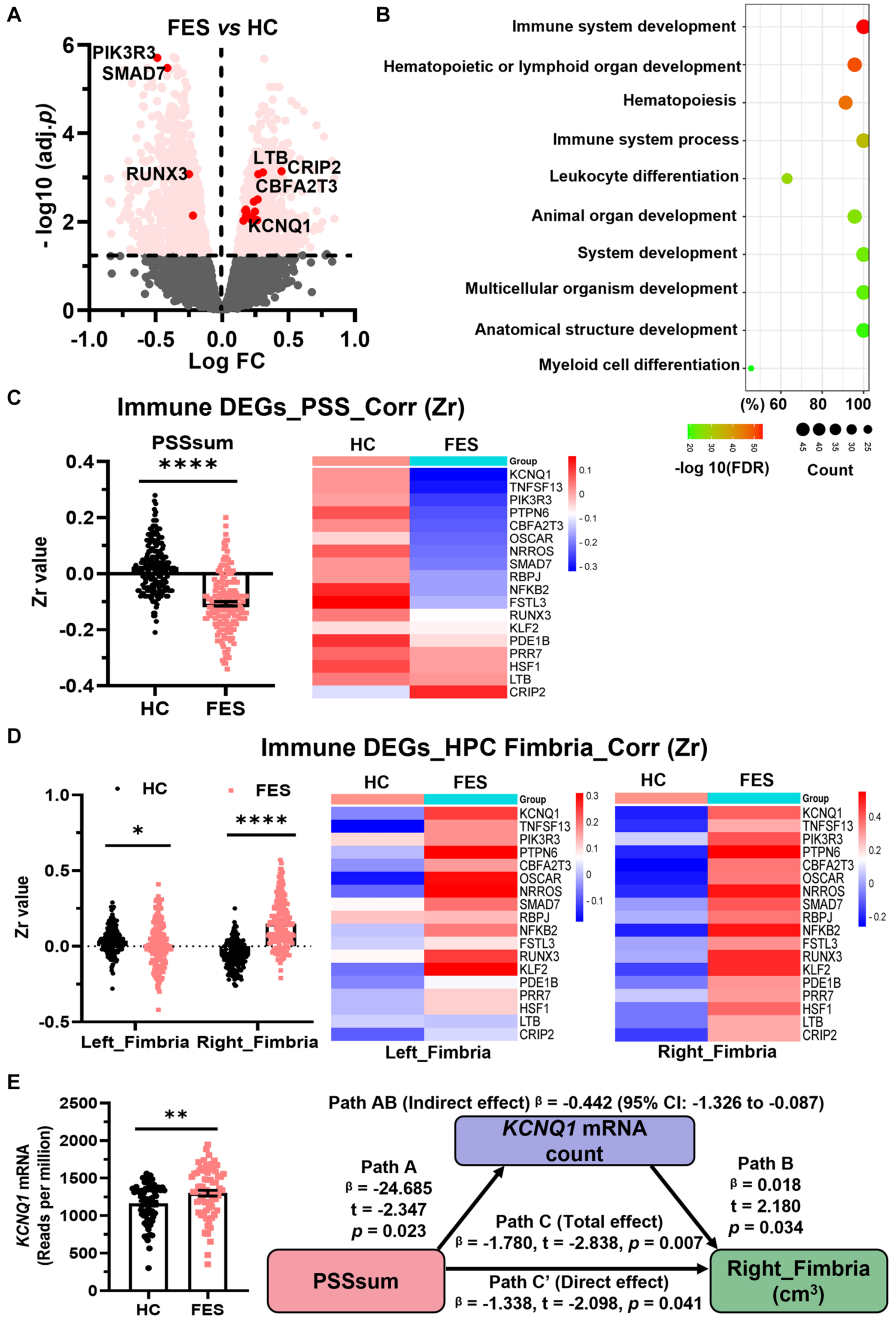
Blood immdev-DEGs were correlated negatively with PSS and positively with the right hippocampal fimbria in FES. **(A)** Volcano plot showing expressions of blood DEGs between FES patients (*n* = 74) and HCs (*n* = 68) after bulk RNA-seq. Pink dots indicate 181 significant DEGs involved in immune system development. Those with values of -log10(FDR) ≥ 2 and also correlated with PSSsum are highlighted in red dots. **(B)** GO-BP enrichment analysis of the 181 immdev-DEGs showing top 10 pathways based on -log10(FDR) clustering values and gene counts. See also Supplementary Table S7. **(C,D)** These 181 immdev-DEGs differed between HC and FES in correlations of their RNAseq counts with PSSsum scores [**(C)**, Spearman’s correlation] and right fimbria volumes [**(D)**, Pearson’s partial correlation controlled for age, sex, and BMI], as represented in dot charts. Correlational coefficients were transformed into Fisher Zr values before comparisons. The top 18 immdev-DEGs with the most significant correlational differences are shown in heat maps. Mean ± SEM; * *p* < 0.05, **** *p* < 0.0001. **(E)** *KCNQ1* mRNA count was elevated in FES compared to HC (** *FDR* < 0.01) and partially mediated negative regulation of PSS on right fimbria volume (Path AB, controlled by age, sex, and BMI). PSS was negatively correlated with *KCNQ1* (Path A, *p* < 0.05), while *KCNQ1* was positively correlated with the right fimbria (Path B, *p* < 0.05). While PSS directly impacted the right fimbria (Path C′, *p* < 0.05), this effect was stronger when *KCNQ1* was considered as a mediator (path C, *p* < 0.01). See also Supplementary Figure S3.

Assuming genes involved in microglial development were similarly altered in CUS-exposed animals and FES patients, we explored blood RNA-seq data on FES patients and HCs. Similar to animal RNA-seq results (Figure 2B), the top GO-BP pathway of human blood DEGs involved nucleotide metabolism and transcription regulation (Supplementary Table S7). Furthermore, we found 181 DEGs involved in immune system development (Figure 6A, Supplementary Table S7), such as hematopoiesis and leukocyte differentiation (Figure 6B). Compared to HCs, these 181 immdev-DEGs overall showed a negative correlation with PSS scores (*p* < 0.0001, Figure 6C and Supplementary Figure S3B) and a positive correlation with hippocampal fimbria volumes, particularly on the right side (*p* < 0.0001, Figure 6D and Supplementary Figure S3B) in FES. Among these immdev-DEGs, 18 genes with the most significant correlational coefficients (Zr) kept such trends in FES, which were reversed in HCs in most cases (Figures 6C,D). We further undertook a mediator analysis assuming stress negatively affected hippocampal structures via certain immdev-DEGs. Notably, *KCNQ1*, encoding a voltage-gated potassium channel, was upregulated in FES patients (*FDR* < 0.01, Figure 6E) similar to CUS + repMg-exposed animals (Supplementary Figure S3A). Furthermore, *KCNQ1* partially mediated the negative regulation of right fimbria volume by PSS (β = −0.442, 95% CI: −1.326 ~ −0.087, Figure 6E).

These data overall mark the right hippocampal fimbria as an important stress-sensitive subregion that immune genes may help regulate in FES, corroborating microglia-mediated brain functions in CUS-devastated animals.

## Discussion

This study aimed to address how microglia may benefit brain and psychiatric behaviors in stressful contexts after they were reprogrammed in animals and whether this may recapitulate the immune mechanism underlying schizophrenia. Our main findings were the following: (1) Microglial renewal partially rescued CUS-triggered deficits in behavior and brain developmental processes such as axonal/synaptic formation modulated by glial cells and (2) FES patients showed higher stress sensitivity along with smaller hippocampal structures and changed expression of genes involved in immune development. Overall, these findings suggest that microglial reprogramming may benefit psychiatric disorders such as schizophrenia.

We found that, after microglial depletion and replenishment, animals showed enhanced anxiety similar to that in CUS condition, and repMg could not rescue CUS-induced anxiety. As anxiety is the foundation for animals to survive and adapt to stress behaviorally, its reset due to repMg is understandable. Notably, CUS-triggered social withdrawal and depression-like behavior were partially or fully recovered after microglial renewal in our experiments. Currently available studies offer mixed results, with some showing the harmful effect of microglial ablation or repopulation on psychiatric-like behaviors (Torres et al., 2016; Rosin et al., 2018; Zhang et al., 2020), while others show no beneficial effect (Elmore et al., 2015; Lehmann et al., 2019; Weber et al., 2019; Wang et al., 2020; Ikezu et al., 2021) in various chronic stress models. This suggests an intricate brain immune environment in psychiatric conditions. Besides, only a few animal stress-modeling papers performed microglial repopulation experiments (Elmore et al., 2015; Rosin et al., 2018; Lehmann et al., 2019; Weber et al., 2019), while many studies did not adopt such an approach (Torres et al., 2016; Sellgren et al., 2019; Wang et al., 2020; Zhang et al., 2020; Ikezu et al., 2021). Thus, our study filled the knowledge gap of how replenished microglia behave in the CUS condition that is highly relevant to schizophrenia.

Our results on flow cytometry showed that mice from the CUS + repMg group had a higher percentage of hippocampal microglia than those of the repMg group, indicating that, in the CUS context, stressed microglia that survived the CSF1Ri-induced ablation may have been more sensitive to the loss of their peers in the neighborhood and may have proliferated or migrated faster to fill the empty space. The CUS-induced inflammatory environment may also have provided proliferative or migratory cues to replenishing microglia. Thus, microglial replenishment may be transiently accelerated by the CUS. It should, however, be pointed out that we only studied microglial replenishment after 14 days of recovery from ablation. It is unclear what would be the long-term effect of CUS on microglial recovery, which is worth addressing in the future. Other studies have shown that chronic stress could increase regional microglial density in IHC, possibly due to increased microglial proliferation and/or accumulation (Tynan et al., 2010; Kreisel et al., 2014). In the present study, we did not observe a gross increase of microglia by CUS alone compared to Ctr, which suggests that the effect may be dependent on the stress model and duration, as has been pointed out by earlier studies (Kreisel et al., 2014; Koo and Wohleb, 2021).

Our result on RNA-seq of the PFC also directed us to investigate microglial synaptic pruning by IHC and flow cytometry. Microglia phagocytose neuronal elements contribute to the structural and functional remodeling of neurons in chronic stress (Andoh and Koyama, 2021). For instance, microglial depletion induced a significant reduction in hippocampal spine density and glutamatergic activity, which recovered after microglial repopulation (Basilico et al., 2022). In our CUS model, stressed microglia showed excessive engulfment of SYP and VGLUT2 along with hyper-ramified morphology, which could be reversed by repopulated microglia. Remarkably, we found that CUS also enhanced VGLUT2 pruning of astrocytes and OPCs, both known important regulators of synaptic or axonal functions in psychiatric disorders (Rial et al., 2015; Dietz et al., 2020; Zhou B. et al., 2021). Attenuation of astrocytic pruning by microglial repopulation after CUS could be due to dampened neuroinflammation, which helps restore glutamate metabolism in astrocytes, as demonstrated previously (Jha et al., 2019). Moreover, microglial repopulation could restore the expression of brain developmental genes changed by CUS. As crosstalk of microglia with astrocytes and oligodendrocytes is important for brain development and plasticity (Jha et al., 2019; Dietz et al., 2020; Zhou B. et al., 2021), repopulated microglia could help in stress resilience of their glial counterparts via, for instance, CSF1R signaling (Marzan et al., 2021).

In FES patients, we observed reduced volumes of hippocampal subregions, such as the fimbria and tails, as compared to HCs, and reduced fimbria sizes were associated with PSS and PANSS scores. Moreover, blood DEGs changed in FES patients, which included those involved in immune system development, and approximately 25% of them were significantly correlated negatively with PSS but positively with the right fimbria. This reveals a close association between the immune and nervous systems that can be impacted by stress during development and suggests that early immune system maldevelopment combined with abnormal brain abnormality may contribute to schizophrenia (Anders and Kinney, 2015; Zengeler and Lukens, 2021). This also corroborates previous evidence on the association between childhood trauma and inflammatory phenotype in schizophrenia patients (Dennison et al., 2012; Chase et al., 2019; Paquin et al., 2021), indicating that immune alterations in patients may be due to stress hypersensitivity caused by early life adversities. Additionally, *KCNQ1*, a known risk gene for schizophrenia (Rannals et al., 2016; Bruce et al., 2017), was elevated in FES patients and CUS + repMg-exposed animals, possibly as a compensational mechanism. Moreover, its correlation with both PSS and fimbria volume implies it acts as a nexus between microglia and stress regulation in schizophrenia. Overall, our results imply that reprogrammed microglia in animals may recapitulate changes in the myeloid developmental process shown in our FES patients, which may negatively impact hippocampal development.

Our study has several limitations, such as the focus on male mice in our preclinical cohort and the cross-sectional nature and small sample size in our clinical cohort. Nevertheless, our study overall supports the idea that microglial replenishment may benefit psychiatric disorders such as schizophrenia.

## Data availability statement

The datasets presented in this study can be found in online repositories. The names of the repository/repositories and accession number(s) can be found at: https://www.ebi.ac.uk/ena, PRJEB53454.

## Ethics statement

The studies involving humans were approved by Institutional Ethical Committee of Beijing Huilongguan Hospital. The studies were conducted in accordance with the local legislation and institutional requirements. Written informed consent for participation in this study was provided by the participants' legal guardians/next of kin. The animal study was approved by Estonian National Board of Animal Experiments. The study was conducted in accordance with the local legislation and institutional requirements.

## Author contributions

LY: Writing – original draft, Data curation, Formal analysis, Investigation. F-LX: Data curation, Formal analysis, Writing – original draft. SC: Writing – review & editing, Investigation, Validation. MG: Writing – review & editing, Investigation, Validation. WC: Writing – review & editing, Investigation. YL: Writing – review & editing, Investigation. ZW: Supervision, Writing – review & editing. LW: Investigation, Writing – review & editing. TX: Investigation, Writing – review & editing. FF: Investigation, Writing – review & editing. AZ: Funding acquisition, Supervision, Writing – review & editing. YT: Funding acquisition, Investigation, Project administration, Resources, Supervision, Writing – review & editing.

## Funding

This work was supported by the National Natural Science Foundation of China (grant nos.: 82001415 and 82171507), the Estonian Research Council-European Union Regional Developmental Fund Mobilitas Plus Program No. MOBTT77, and Personal Research Funding Team (grant no. PRG878).

## Conflict of interest

The authors declare that the research was conducted in the absence of any commercial or financial relationships that could be construed as a potential conflict of interest.

## Publisher’s note

All claims expressed in this article are solely those of the authors and do not necessarily represent those of their affiliated organizations, or those of the publisher, the editors and the reviewers. Any product that may be evaluated in this article, or claim that may be made by its manufacturer, is not guaranteed or endorsed by the publisher.

## Figures and Tables

**Table:** 

ANOVA	analysis of variance
CUS	chronic unpredictable stress
CSF1Ri	colony-stimulating factor 1 receptor inhibitor
Ctr	control
CTQsum	childhood trauma scale summation
DEGs	differentially expressed genes
EPM	elevated plus maze
FES	first episode schizophrenia
FDR	false discovery rate
HC	healthy control
MRI	Magnetic resonance imaging
OFT	open field test
PANSS	positive and negative symptom scale
PANSSN	negative scale of PANSS
PANSSP	positive scale of PANSS
PANSSG	general psychopathology scores of PANSS
PANSST	total scores of PANSS
PFA	paraformaldehyde
PFC	prefrontal cortex
PSSsum	perceived stress scale summation
RepMg	microglial repopulation
RNA-seq	RNA sequencing
SYP	synaptophysin
TCT	three-chamber test
TST	tail suspension test
VGLUT2	vesicular glutamate transporter 2

## Footnotes

^1^ https://david.ncifcrf.gov/

^2^ https://atlas.brain-map.org/

^3^ http://www.bioinformatics.com.cn/
