# Supplementary material for: Replenished microglia partially rescue schizophrenia-related stress response
Source: Front Cell Neurosci. 2023 Sep 12;17:1254923. doi: 10.3389/fncel.2023.1254923 (PMC10522857; doi:10.3389/fncel.2023.1254923)
Supplement: Supplementary file 1 [file Data_Sheet_1.PDF]

Table S1: GOBP pathways of mouse PFC DEGs after CUS and repMg treatments

Category	Term	Count	%	PValue	Genes
GOTERM_BP_DIRECT	GO:0045944~positive regulation of transcription from RNA polymerase II promoter	97	9,082397	2,39175E-07	PRDM2, RC
GOTERM_BP_DIRECT	GO:0000122~negative regulation of transcription from RNA polymerase II promote	80	7,490637	1,093E-06	ZFP169, EH
GOTERM_BP_DIRECT	GO:0007399~nervous system development	45	4,213483	1,46772E-06	ROBO3, HD
GOTERM_BP_DIRECT	GO:0007411~axon guidance	25	2,340824	7,40062E-06	MEG3, ROE
GOTERM_BP_DIRECT	GO:0045893~positive regulation of transcription, DNA-templated	60	5,617978	1,33301E-05	PHF20, ROI
GOTERM_BP_DIRECT	GO:0034765~regulation of ion transmembrane transport	20	1,872659	4,48208E-05	KCNG1, HC
GOTERM_BP_DIRECT	GO:0007156~homophilic cell adhesion via plasma membrane adhesion molecules	22	2,059925	5,27074E-05	PTPRT, PCD
GOTERM_BP_DIRECT	GO:0006468~protein phosphorylation	54	5,05618	6,80137E-05	PRR5, MAS
GOTERM_BP_DIRECT	GO:0007275~multicellular organism development	80	7,490637	6,99841E-05	ROBO3, RY
GOTERM_BP_DIRECT	GO:0001701~in utero embryonic development	34	3,183521	8,64802E-05	MEG3, TSH

List Total	Pop Hits	Pop Total	Fold Enricl	Bonferroni	Benjamini	FDR
937	1216	20094	1,710665	0,000946	0,000947	0,000944
937	975	20094	1,759593	0,004318	0,001937	0,001931
937	438	20094	2,203257	0,005794	0,001937	0,001931
937	188	20094	2,851734	0,028874	0,007325	0,007301
937	712	20094	1,807166	0,051406	0,010555	0,01052
937	145	20094	2,957936	0,162597	0,029574	0,029477
937	172	20094	2,74297	0,18834	0,02981	0,029712
937	655	20094	1,767988	0,236067	0,030785	0,030684
937	1095	20094	1,566761	0,242004	0,030785	0,030684
937	347	20094	2,101243	0,289929	0,034238	0,034125

Table S2: GOBP pathways of mouse PFC DEGs after repMg treatment

Category	Term	Count	%	PValue	Genes
GOTERM_BP_DIRECT	GO:0045944~positive regulation of transcription from RNA polymerase II promoter	139	8,764187	2,94483E-10	RB1, GABPI
GOTERM_BP_DIRECT	GO:0006357~regulation of transcription from RNA polymerase II promoter	169	10,65574	1,6181E-09	RB1, ZFP44
GOTERM_BP_DIRECT	GO:0000122~negative regulation of transcription from RNA polymerase II promoter	111	6,998739	2,94183E-08	RB1, ZFP16
GOTERM_BP_DIRECT	GO:0045892~negative regulation of transcription, DNA-templated	81	5,107188	1,50588E-07	RB1, NAB2,
GOTERM_BP_DIRECT	GO:0048511~rhythmic process	30	1,891551	2,7159E-07	KDM5A, PR
GOTERM_BP_DIRECT	GO:0032922~circadian regulation of gene expression	18	1,134931	3,66371E-06	KDM5A, EG
GOTERM_BP_DIRECT	GO:0045893~positive regulation of transcription, DNA-templated	78	4,918033	1,70078E-05	KDM5A, TR
GOTERM_BP_DIRECT	GO:0035335~peptidyl-tyrosine dephosphorylation	12	0,75662	1,8664E-05	PTPRT, DUS
GOTERM_BP_DIRECT	GO:0048167~regulation of synaptic plasticity	15	0,945776	1,99419E-05	BDNF, BAIA
GOTERM_BP_DIRECT	GO:0000188~inactivation of MAPK activity	8	0,504414	2,28613E-05	SPRED3, DU
GOTERM_BP_DIRECT	GO:0030336~negative regulation of cell migration	26	1,639344	2,98031E-05	PTPRT, KAN
GOTERM_BP_DIRECT	GO:0030512~negative regulation of transforming growth factor beta receptor signaling p	19	1,197982	3,08744E-05	XBP1, FAM
GOTERM_BP_DIRECT	GO:0001822~kidney development	28	1,765448	3,69951E-05	CFH, SCHIP
GOTERM_BP_DIRECT	GO:0006468~protein phosphorylation	71	4,476671	6,01013E-05	DYRK3, BM
GOTERM_BP_DIRECT	GO:0045664~regulation of neuron differentiation	12	0,75662	8,67501E-05	EFNA3, ZFH
GOTERM_BP_DIRECT	GO:0007507~heart development	39	2,459016	8,93207E-05	HDAC5, CD
GOTERM_BP_DIRECT	GO:0045332~phospholipid translocation	10	0,630517	9,97306E-05	ABCA1, ATI
GOTERM_BP_DIRECT	GO:0042752~regulation of circadian rhythm	15	0,945776	0,000129634	PRKCG, KLF

List Total	Pop Hits	Pop Total	Fold Enricl	Bonferroni	Benjamini	FDR
1339	1216	20094	1,715406	1,45E-06	1,45E-06	1,44E-06
1339	1606	20094	1,579163	7,97E-06	3,99E-06	3,97E-06
1339	975	20094	1,708458	0,000145	4,83E-05	4,81E-05
1339	662	20094	1,83617	0,000742	0,000186	0,000185
1339	153	20094	2,942494	0,001338	0,000268	0,000266
1339	72	20094	3,75168	0,017893	0,003009	0,002994
1339	712	20094	1,643995	0,080399	0,010919	0,010864
1339	37	20094	4,867045	0,087874	0,010919	0,010864
1339	58	20094	3,881049	0,0936	0,010919	0,010864
1339	15	20094	8,003585	0,106547	0,011266	0,011209
1339	154	20094	2,533602	0,136595	0,012679	0,012615
1339	92	20094	3,099214	0,141142	0,012679	0,012615
1339	175	20094	2,401075	0,166661	0,014024	0,013953
1339	655	20094	1,626683	0,256352	0,021156	0,021048
1339	43	20094	4,187922	0,347878	0,027511	0,027371
1339	298	20094	1,963967	0,356087	0,027511	0,027371
1339	30	20094	5,00224	0,38829	0,02891	0,028763
1339	68	20094	3,310306	0,472114	0,035491	0,035311

Table S3: GOBP pathways of mouse PFC DEGs after CUS treatment

Category	Term	Count	%	PValue	Genes
GOTERM_BP_DIRECT	GO:0034976~response to endoplasmic reticulum stress	6	5,454545	6,35E-05	XBP1, SDF
GOTERM_BP_DIRECT	GO:0042738~exogenous drug catabolic process	5	4,545455	0,00011	CYP2A5, C
GOTERM_BP_DIRECT	GO:0019373~epoxygenase P450 pathway	4	3,636364	0,000725	CYP2A5, C
GOTERM_BP_DIRECT	GO:0006805~xenobiotic metabolic process	5	4,545455	0,001109	CYP2A5, C
GOTERM_BP_DIRECT	GO:0050911~detection of chemical stimulus involved in sensory perception of sm	4	3,636364	0,001129	OLFR1507,
GOTERM_BP_DIRECT	GO:0006082~organic acid metabolic process	4	3,636364	0,001853	CYP2A5, C
GOTERM_BP_DIRECT	GO:0001525~angiogenesis	7	6,363636	0,002397	TBX1, XBP
GOTERM_BP_DIRECT	GO:0006629~lipid metabolic process	11	10	0,002462	XBP1, SLC
GOTERM_BP_DIRECT	GO:0017144~drug metabolic process	3	2,727273	0,004041	AOX2, CYP
GOTERM_BP_DIRECT	GO:0008202~steroid metabolic process	5	4,545455	0,004653	SRD5A2, I
GOTERM_BP_DIRECT	GO:0071353~cellular response to interleukin-4	3	2,727273	0,007306	XBP1, HSP
GOTERM_BP_DIRECT	GO:0070373~negative regulation of ERK1 and ERK2 cascade	4	3,636364	0,007822	DUSP4, SP
GOTERM_BP_DIRECT	GO:0042632~cholesterol homeostasis	4	3,636364	0,012167	XBP1, CES
GOTERM_BP_DIRECT	GO:0045944~positive regulation of transcription from RNA polymerase II promote	13	11,81818	0,012784	TBX1, XBP
GOTERM_BP_DIRECT	GO:0018894~dibenzo-p-dioxin metabolic process	2	1,818182	0,014235	SRD5A2, C
GOTERM_BP_DIRECT	GO:0001569~branching involved in blood vessel morphogenesis	3	2,727273	0,016347	TBX1, COL
GOTERM_BP_DIRECT	GO:0006351~transcription, DNA-templated	4	3,636364	0,016464	COL4A2, K
GOTERM_BP_DIRECT	GO:0008203~cholesterol metabolic process	4	3,636364	0,018062	CES1D, INS
GOTERM_BP_DIRECT	GO:0006706~steroid catabolic process	2	1,818182	0,023614	SRD5A2, C
GOTERM_BP_DIRECT	GO:0006366~transcription from RNA polymerase II promoter	5	4,545455	0,026809	XBP1, LEF1
GOTERM_BP_DIRECT	GO:0030540~female genitalia development	2	1,818182	0,02827	SRD5A2, T
GOTERM_BP_DIRECT	GO:0071499~cellular response to laminar fluid shear stress	2	1,818182	0,02827	XBP1, KLF
GOTERM_BP_DIRECT	GO:1903895~negative regulation of IRE1-mediated unfolded protein response	2	1,818182	0,02827	HSPA5, PD
GOTERM_BP_DIRECT	GO:0006986~response to unfolded protein	3	2,727273	0,03228	XBP1, HSP
GOTERM_BP_DIRECT	GO:0048752~semicircular canal morphogenesis	2	1,818182	0,037517	TBX1, TBX
GOTERM_BP_DIRECT	GO:0000122~negative regulation of transcription from RNA polymerase II promot	10	9,090909	0,042878	TBX1, KLF1
GOTERM_BP_DIRECT	GO:0016098~monoterpenoid metabolic process	2	1,818182	0,046676	CYP1A2, C
GOTERM_BP_DIRECT	GO:0042472~inner ear morphogenesis	3	2,727273	0,048682	TBX1, INS

List Total	Pop Hits	Pop Total	Fold Enricl	Bonferron	Benjamini	FDR
97	88	20136	14,1537	0,052343	0,046619	0,046399
97	52	20136	19,96035	0,089028	0,046619	0,046399
97	37	20136	22,44191	0,458794	0,190968	0,190065
97	95	20136	10,92566	0,608735	0,190968	0,190065
97	43	20136	19,31048	0,615333	0,190968	0,190065
97	51	20136	16,28138	0,791739	0,260323	0,259092
97	284	20136	5,116596	0,868695	0,260323	0,259092
97	728	20136	3,136626	0,875712	0,260323	0,259092
97	20	20136	31,13814	0,967481	0,379888	0,378092
97	141	20136	7,361263	0,980657	0,393625	0,391764
97	27	20136	23,06529	0,997978	0,551422	0,548814
97	85	20136	9,76883	0,998697	0,551422	0,548814
97	100	20136	8,303505	0,999968	0,772542	0,768889
97	1216	20136	2,219276	0,999981	0,772542	0,768889
97	3	20136	138,3918	0,999995	0,802875	0,799078
97	41	20136	15,18934	0,999999	0,819339	0,815465
97	112	20136	7,413844	0,999999	0,819339	0,815465
97	116	20136	7,158194	1	0,848935	0,844921
97	5	20136	83,03505	1	1	0,99645
97	237	20136	4,379486	1	1	0,99645
97	6	20136	69,19588	1	1	0,99645
97	6	20136	69,19588	1	1	0,99645
97	6	20136	69,19588	1	1	0,99645
97	59	20136	10,5553	1	1	0,99645
97	8	20136	51,89691	1	1	0,99645
97	975	20136	2,129104	1	1	0,99645
97	10	20136	41,51753	1	1	0,99645
97	74	20136	8,415715	1	1	0,99645

Table S4. Hippocampal associations with PSSsum in FES patients and HCs

Cortical regions	FES-PSS (*n*=54)		HC-PSS (*n*=64)	
	r	***p***	***r***	***p***
HP	-0.151	0.276	**0.276**	**0.028**
Left_Whole HP	-0.020	0.885	**0.271**	**0.031**
Right_Whole HP	-0.234	0.089	**0.274**	**0.028**
Left_CA1	0.043	0.759	0.200	0.114
Left_CA3	0.046	0.741	0.183	0.147
Left_CA4	0.036	0.794	**0.249**	**0.047**
Left_DG	0.017	0.904	0.234	0.062
Left_Fimbria	-0.256	0.061	0.093	0.464
Left_Fissure	-0.032	0.818	0.160	0.206
Left_HATA	-0.109	0.433	0.056	0.658
Left_Molecular layer	0.038	0.783	**0.259**	**0.039**
Left_Parasubiculum	0.192	0.165	0.221	0.079
Left_Presubiculum	0.176	0.203	**0.276**	**0.028**
Left_Subiculum	-0.074	0.593	0.226	0.073
Left_Tail	-0.059	0.672	0.185	0.144
Right_CA1	**-0.276**	**0.044**	0.213	0.091
Right_CA3	-0.220	0.111	0.226	0.072
Right_CA4	-0.217	0.116	**0.270**	**0.031**
Right_DG	-0.216	0.117	**0.253**	**0.044**
Right_Fimbria	**-0.397**	**0.003**	0.060	0.636
Right_Fissure	0.060	0.666	0.039	0.762
Right_HATA	-0.202	0.143	0.168	0.184
Right_Molecular layer	**-0.299**	**0.028**	**0.303**	**0.015**
Right_Parasubiculum	0.074	0.595	0.043	0.738
Right_Presubiculum	-0.001	0.992	0.176	0.165
Right_Subiculum	-0.222	0.106	**0.325**	**0.009**
Right_Tail	0.028	0.839	0.216	0.086

Spearman's correlation

Table S5. Hippocampal associations with PANSS in FES patients (*n*=51)

Cortical regions [1]	PANSSP	PANSSN	PANSSG	PANSST

HP	***r***	-0.093	0.103	-0.032	-0.004
	p	0.509	0.462	0.819	0.977
Left_Whole_HP	***r***	-0.149	0.075	-0.132	-0.094
	p	0.287	0.595	0.344	0.502
Right_Whole_HP	***r***	-0.062	0.056	0.029	0.022
	p	0.657	0.693	0.838	0.876
Left_CA1	***r***	-0.269	-0.008	**-0.292**	-0.263
	p	0.052	0.956	**0.034**	0.057
Left_CA3	***r***	-0.269	0.005	-0.241	-0.230
	p	0.052	0.969	0.082	0.098
Left_CA4	***r***	-0.084	-0.015	-0.144	-0.118
	p	0.550	0.914	0.302	0.402
Left_DG	***r***	-0.169	-0.015	-0.149	-0.148
	p	0.226	0.917	0.285	0.289
Left_Fimbria	***r***	-0.186	**-0.388**	-0.200	**-0.339**
	p	0.182	**0.004**	0.151	**0.013**
Left_Fissure	***r***	-0.061	0.139	-0.090	-0.015
	p	0.666	0.321	0.521	0.913
Left_HATA	***r***	-0.089	-0.201	-0.226	-0.245
	p	0.528	0.150	0.104	0.077
Left_Molecular layer	***r***	-0.193	0.106	-0.140	-0.101
	p	0.167	0.450	0.316	0.473
Left_Parasubiculum	***r***	-0.012	0.124	0.056	0.081
	p	0.934	0.378	0.693	0.563
Left_Presubiculum	***r***	-0.088	**0.272**	0.103	0.146
	p	0.529	**0.049**	0.465	0.296
Left_Subiculum	***r***	0.042	0.036	0.037	0.054
	p	0.767	0.798	0.793	0.703
Left_Tail	***r***	0.117	0.129	0.047	0.121
	p	0.405	0.357	0.741	0.390
Right_CA1	***r***	-0.104	-0.091	-0.078	-0.116
	p	0.459	0.516	0.581	0.410
Right _CA3	***r***	-0.155	-0.225	-0.118	-0.215
	p	0.267	0.106	0.399	0.122
Right _CA4	***r***	-0.111	-0.188	-0.100	-0.172
	p	0.428	0.179	0.477	0.217
Right _DG	***r***	-0.200	-0.162	-0.147	-0.219
	p	0.150	0.247	0.293	0.115
Right _Fimbria	***r***	-0.040	-0.155	-0.153	-0.168
	p	0.776	0.269	0.273	0.231

Right _Fissure	***r***	-0.144	0.177	0.053	0.059
	p	0.305	0.206	0.709	0.676
Right _HATA	***r***	-0.159	0.124	-0.091	-0.056
	p	0.256	0.378	0.518	0.690
Right _Molecular layer	***r***	-0.042	-0.033	0.017	-0.015
	p	0.763	0.813	0.906	0.912
Right _Parasubiculum	***r***	-0.057	**0.374**	0.092	0.190
	p	0.684	**0.006**	0.515	0.173
Right _Presubiculum	***r***	-0.042	**0.320**	0.182	0.229
	p	0.767	**0.020**	0.191	0.099
Right _Subiculum	***r***	-0.018	0.154	0.093	0.115
	p	0.898	0.272	0.509	0.411
Right_Tail	***r***	0.133	0.214	0.172	0.237
	p	0.341	0.124	0.217	0.088

[1]Analysis of covariance with age, sex and BMI as covariates.

Table S6. Hippocampal associations with CTQ in FES patients and HCs

**Cortical regions**	**FES-CTQ (*n*=48)**		**HC-CTQ (*n*=58)**	
	r	***p***	***r***	***p***
HP	0.228	0.119	0.018	0.896
Left_Whole HP	0.137	0.353	0.106	0.428
Right_Whole HP	0.112	0.448	0.032	0.813
Left_CA1	0.123	0.405	0.132	0.322
Left_CA3	-0.187	0.203	0.140	0.295
Left_CA4	-0.095	0.521	0.197	0.138
Left_DG	-0.058	0.697	0.200	0.132
Left_Fimbria	-0.160	0.276	0.003	0.984
Left_Fissure	0.199	0.176	-0.167	0.210
Left_HATA	-0.146	0.322	0.070	0.600
Left_Molecular layer	0.098	0.507	0.106	0.428
Left_Parasubiculum	-0.073	0.622	0.154	0.248
Left_Presubiculum	0.156	0.289	0.006	0.962
Left_Subiculum	0.216	0.289	-0.010	0.941
Left_Tail	0.185	0.208	-0.03	0.821
Right_CA1	0.085	0.566	0.024	0.859
Right_CA3	0.000	0.999	0.075	0.575

Right_CA4	-0.024	0.869	0.052	0.696
Right_DG	-0.034	0.819	0.030	0.821
Right_Fimbria	-0.039	0.795	0.050	0.711
Right_Fissure	0.028	0.848	-0.089	0.506
Right_HATA	0.006	0.968	-0.043	0.751
Right_Molecular layer	0.052	0.727	0.060	0.653
Right_Parasubiculum	-0.007	0.964	0.056	0.677
Right_Presubiculum	0.166	0.260	0.062	0.645
Right_Subiculum	0.173	0.239	0.177	0.183
Right_Tail	0.205	0.162	-0.113	0.397

Spearman's correlation

Table S7: GOBP pathways of blood DEGs in human FES patients

Category	Term	Count	%	PValue	Genes
GOTERM_BP_ALL	GO:0019219~regulation of nucleobase-containing compound metabolic process	679	25,94574	1,45E-16	ATF1, RB1,
GOTERM_BP_ALL	GO:0043412~macromolecule modification	669	25,56362	3,22E-16	RB1, ATF2,
GOTERM_BP_ALL	GO:0036211~protein modification process	638	24,37906	5,38E-16	RB1, ATF2,
GOTERM_BP_ALL	GO:0006464~cellular protein modification process	638	24,37906	5,38E-16	RB1, ATF2,
GOTERM_BP_ALL	GO:0065009~regulation of molecular function	533	20,36683	7,14E-16	RB1, ATF2,
GOTERM_BP_ALL	GO:0051252~regulation of RNA metabolic process	618	23,61483	3,51E-15	ATF1, RB1,
GOTERM_BP_ALL	GO:0006351~transcription, DNA-templated	585	22,35384	3,93E-15	ATF1, RB1,
GOTERM_BP_ALL	GO:0034654~nucleobase-containing compound biosynthetic process	669	25,56362	5,39E-15	ATF1, RB1,
GOTERM_BP_ALL	GO:0032774~RNA biosynthetic process	607	23,1945	1,23E-14	ATF1, RB1,
GOTERM_BP_ALL	GO:2001141~regulation of RNA biosynthetic process	587	22,43026	1,55E-14	ATF1, RB1,
GOTERM_BP_ALL	GO:0006355~regulation of transcription, DNA-templated	583	22,27742	3,02E-14	ATF1, RB1,
GOTERM_BP_ALL	GO:0097659~nucleic acid-templated transcription	601	22,96523	3,23E-14	ATF1, RB1,
GOTERM_BP_ALL	GO:1903506~regulation of nucleic acid-templated transcription	583	22,27742	3,32E-14	ATF1, RB1,
GOTERM_BP_ALL	GO:0019438~aromatic compound biosynthetic process	675	25,79289	4,34E-14	ATF1, RB1,
GOTERM_BP_ALL	GO:0018130~heterocycle biosynthetic process	673	25,71647	6,47E-14	ATF1, RB1,
GOTERM_BP_ALL	GO:1901362~organic cyclic compound biosynthetic process	690	26,36607	2,55E-13	ATF1, RB1,
GOTERM_BP_ALL	GO:0050790~regulation of catalytic activity	421	16,08712	3,27E-13	RB1, GMFI
GOTERM_BP_ALL	GO:0006357~regulation of transcription from RNA polymerase II promoter	451	17,23347	7,09E-13	ATF1, RB1,
GOTERM_BP_ALL	GO:0065007~biological regulation	1758	67,17616	1,15E-12	ATF1, ATF
GOTERM_BP_ALL	GO:0051641~cellular localization	485	18,53267	1,29E-12	RB1, ATF2,
GOTERM_BP_ALL	GO:0050794~regulation of cellular process	1590	60,75659	5,54E-12	ATF1, ATF
GOTERM_BP_ALL	GO:0050789~regulation of biological process	1666	63,66068	6,28E-12	ATF1, ATF
GOTERM_BP_ALL	GO:0048518~positive regulation of biological process	946	36,14826	1,43E-11	ATF1, ATF
GOTERM_BP_ALL	GO:0048522~positive regulation of cellular process	862	32,93848	1,69E-11	ATF1, ATF
GOTERM_BP_ALL	GO:0035556~intracellular signal transduction	441	16,85136	4,72E-11	ATF1, RB1,
GOTERM_BP_ALL	GO:0031325~positive regulation of cellular metabolic process	557	21,28391	9,61E-11	ATF1, RB1,
GOTERM_BP_ALL	GO:0008104~protein localization	422	16,12533	1,45E-10	RB1, ATF2,
GOTERM_BP_ALL	GO:0031323~regulation of cellular metabolic process	987	37,71494	1,72E-10	ATF1, ATF
GOTERM_BP_ALL	GO:0033554~cellular response to stress	342	13,0684	2E-10	ATF2, SPI1
GOTERM_BP_ALL	GO:0045934~negative regulation of nucleobase-containing compound metabolic process	282	10,7757	2,16E-10	IFITM3, RE
GOTERM_BP_ALL	GO:0051649~establishment of localization in cell	364	13,90906	2,32E-10	ATF2, IPO1

GOTERM_BP_ALL	GO:0009893~positive regulation of metabolic process	591	22,58311	2,49E-10	ATF1, RB1,
GOTERM_BP_ALL	GO:0019222~regulation of metabolic process	1047	40,00764	2,71E-10	ATF1, ATF
GOTERM_BP_ALL	GO:0006366~transcription from RNA polymerase II promoter	347	13,25946	3,1E-10	RB1, ATF1,
GOTERM_BP_ALL	GO:0048583~regulation of response to stimulus	647	24,72297	5,65E-10	RB1, PGLY
GOTERM_BP_ALL	GO:0051253~negative regulation of RNA metabolic process	257	9,820405	6,55E-10	IFITM3, RB
GOTERM_BP_ALL	GO:0033036~macromolecule localization	473	18,07413	9,22E-10	RB1, ATF2,
GOTERM_BP_ALL	GO:0080090~regulation of primary metabolic process	964	36,83607	9,81E-10	ATF1, ATF
GOTERM_BP_ALL	GO:0065008~regulation of biological quality	619	23,65304	1,61E-09	RB1, ATF2,
GOTERM_BP_ALL	GO:0010604~positive regulation of macromolecule metabolic process	543	20,74895	1,78E-09	ATF1, RB1,
GOTERM_BP_ALL	GO:1902679~negative regulation of RNA biosynthetic process	245	9,361865	1,78E-09	IFITM3, RB
GOTERM_BP_ALL	GO:0046907~intracellular transport	295	11,27245	2,36E-09	ATF2, IPO1
GOTERM_BP_ALL	GO:0045892~negative regulation of transcription, DNA-templated	241	9,209018	2,77E-09	RB1, ATF2,
GOTERM_BP_ALL	GO:1903507~negative regulation of nucleic acid-templated transcription	241	9,209018	3,4E-09	RB1, ATF2,
GOTERM_BP_ALL	GO:0016310~phosphorylation	376	14,3676	3,57E-09	EPHB6, RB
GOTERM_BP_ALL	GO:0045184~establishment of protein localization	311	11,88384	6,88E-09	ATF2, IPO1
GOTERM_BP_ALL	GO:0000122~negative regulation of transcription from RNA polymerase II promoter	180	6,878105	8,34E-09	RB1, ATF2,
GOTERM_BP_ALL	GO:0009966~regulation of signal transduction	484	18,49446	8,4E-09	RB1, SPI1,
GOTERM_BP_ALL	GO:0070727~cellular macromolecule localization	317	12,11311	1,76E-08	RB1, ATF2,
GOTERM_BP_ALL	GO:0034613~cellular protein localization	315	12,03668	1,84E-08	RB1, ATF2,
GOTERM_BP_ALL	GO:0045935~positive regulation of nucleobase-containing compound metabolic process	334	12,76271	2,2E-08	RB1, ATF1,
GOTERM_BP_ALL	GO:0051173~positive regulation of nitrogen compound metabolic process	359	13,718	2,29E-08	RB1, ATF1,
GOTERM_BP_ALL	GO:0060255~regulation of macromolecule metabolic process	963	36,79786	2,56E-08	ATF1, ATF
GOTERM_BP_ALL	GO:0051338~regulation of transferase activity	181	6,916316	2,56E-08	EPHB6, RB
GOTERM_BP_ALL	GO:0006468~protein phosphorylation	314	11,99847	2,71E-08	EPHB6, RB
GOTERM_BP_ALL	GO:0006996~organelle organization	618	23,61483	3,86E-08	RB1, PI4K2
GOTERM_BP_ALL	GO:0007049~cell cycle	282	10,7757	3,88E-08	RB1, ATF2,
GOTERM_BP_ALL	GO:0010646~regulation of cell communication	532	20,32862	3,88E-08	RB1, SPI1,
GOTERM_BP_ALL	GO:0023051~regulation of signaling	535	20,44326	4,71E-08	RB1, SPI1,
GOTERM_BP_ALL	GO:0016043~cellular component organization	951	36,33932	5,5E-08	ATF1, ATF
GOTERM_BP_ALL	GO:1902531~regulation of intracellular signal transduction	309	11,80741	8,06E-08	SPI1, STM
GOTERM_BP_ALL	GO:0002520~immune system development	181	6,916316	9,3E-08	RB1, ATF2,
GOTERM_BP_ALL	GO:0006796~phosphate-containing compound metabolic process	482	18,41804	9,69E-08	RB1, PI4K2
GOTERM_BP_ALL	GO:0070647~protein modification by small protein conjugation or removal	187	7,145587	1,05E-07	OTUD4, O

GOTERM_BP_ALL	GO:0006793~phosphorus metabolic process	493	18,83836	1,37E-07	RB1, PI4K2
GOTERM_BP_ALL	GO:0016567~protein ubiquitination	154	5,884601	1,38E-07	FBXO28, L
GOTERM_BP_ALL	GO:0051716~cellular response to stimulus	1053	40,23691	1,47E-07	ATF1, ATF2
GOTERM_BP_ALL	GO:0051171~regulation of nitrogen compound metabolic process	729	27,85632	1,86E-07	ATF1, RB1,
GOTERM_BP_ALL	GO:0031396~regulation of protein ubiquitination	60	2,292702	1,94E-07	GSK3A, PR
GOTERM_BP_ALL	GO:0009889~regulation of biosynthetic process	712	27,20673	1,98E-07	ATF1, RB1,
GOTERM_BP_ALL	GO:0031399~regulation of protein modification process	310	11,84562	2,07E-07	RB1, ATF2,
GOTERM_BP_ALL	GO:0042886~amide transport	299	11,4253	2,35E-07	ATF2, IPO1
GOTERM_BP_ALL	GO:0071840~cellular component organization or biogenesis	969	37,02713	2,53E-07	ATF1, ATF2
GOTERM_BP_ALL	GO:0051254~positive regulation of RNA metabolic process	291	11,1196	2,68E-07	RB1, ATF1,
GOTERM_BP_ALL	GO:0015833~peptide transport	293	11,19603	3,05E-07	ATF2, IPO1
GOTERM_BP_ALL	GO:0030030~cell projection organization	257	9,820405	3,08E-07	EPHB6, RB
GOTERM_BP_ALL	GO:0009057~macromolecule catabolic process	220	8,406572	3,1E-07	PGLYRP2,
GOTERM_BP_ALL	GO:0015031~protein transport	285	10,89033	3,41E-07	ATF2, IPO1
GOTERM_BP_ALL	GO:1903320~regulation of protein modification by small protein conjugation or removal	66	2,521972	3,57E-07	OTUD4, G
GOTERM_BP_ALL	GO:0051603~proteolysis involved in cellular protein catabolic process	143	5,464272	3,96E-07	UBXN2A, L
GOTERM_BP_ALL	GO:0051128~regulation of cellular component organization	430	16,43103	4,29E-07	ATF1, RB1,
GOTERM_BP_ALL	GO:0048534~hematopoietic or lymphoid organ development	169	6,457776	4,95E-07	RB1, ATF2,
GOTERM_BP_ALL	GO:0006139~nucleobase-containing compound metabolic process	889	33,97019	5,12E-07	ATF1, ATF2
GOTERM_BP_ALL	GO:0009891~positive regulation of biosynthetic process	341	13,03019	5,51E-07	RB1, ATF1,
GOTERM_BP_ALL	GO:0032446~protein modification by small protein conjugation	161	6,152083	5,89E-07	FBXO28, L
GOTERM_BP_ALL	GO:0010557~positive regulation of macromolecule biosynthetic process	316	12,07489	5,98E-07	RB1, ATF1,
GOTERM_BP_ALL	GO:0043549~regulation of kinase activity	155	5,922812	6,28E-07	EPHB6, RB
GOTERM_BP_ALL	GO:0010556~regulation of macromolecule biosynthetic process	674	25,75468	7,03E-07	ATF1, RB1,
GOTERM_BP_ALL	GO:0030097~hemopoiesis	162	6,190294	7,88E-07	RB1, ATF2,
GOTERM_BP_ALL	GO:0080134~regulation of response to stress	265	10,1261	9,77E-07	RB1, SPI1,
GOTERM_BP_ALL	GO:0045859~regulation of protein kinase activity	138	5,273214	9,9E-07	RB1, SLC2
GOTERM_BP_ALL	GO:0006974~cellular response to DNA damage stimulus	160	6,113871	1,03E-06	ATF2, DCL
GOTERM_BP_ALL	GO:0090304~nucleic acid metabolic process	809	30,91326	1,04E-06	ATF1, ATF2
GOTERM_BP_ALL	GO:0043085~positive regulation of catalytic activity	217	8,291937	1,15E-06	EPHB6, RA
GOTERM_BP_ALL	GO:1902680~positive regulation of RNA biosynthetic process	277	10,58464	1,16E-06	RB1, ATF1,
GOTERM_BP_ALL	GO:0030163~protein catabolic process	176	6,725258	1,18E-06	UBXN2A, L
GOTERM_BP_ALL	GO:1901576~organic substance biosynthetic process	923	35,26939	1,22E-06	ATF1, ATF2

GOTERM_BP_ALL	GO:0071705~nitrogen compound transport	339	12,95376	1,29E-06	ATF2, IPO1
GOTERM_BP_ALL	GO:0009058~biosynthetic process	936	35,76614	1,44E-06	ATF1, ATF2
GOTERM_BP_ALL	GO:0031326~regulation of cellular biosynthetic process	696	26,59534	1,45E-06	ATF1, RB1,
GOTERM_BP_ALL	GO:0046483~heterocycle metabolic process	906	34,61979	1,53E-06	ATF1, ATF2
GOTERM_BP_ALL	GO:0007264~small GTPase mediated signal transduction	90	3,439052	1,59E-06	RB1, ITGB1
GOTERM_BP_ALL	GO:0006950~response to stress	601	22,96523	1,9E-06	RB1, ATF2,
GOTERM_BP_ALL	GO:0002697~regulation of immune effector process	89	3,400841	2,01E-06	DENND1B,
GOTERM_BP_ALL	GO:0031328~positive regulation of cellular biosynthetic process	331	12,64807	2,07E-06	RB1, ATF1,
GOTERM_BP_ALL	GO:0006511~ubiquitin-dependent protein catabolic process	124	4,73825	2,56E-06	UBXN2A, L
GOTERM_BP_ALL	GO:0071702~organic substance transport	394	15,05541	2,62E-06	ATF2, IPO1
GOTERM_BP_ALL	GO:0080135~regulation of cellular response to stress	146	5,578907	2,93E-06	SPI1, ANKL
GOTERM_BP_ALL	GO:0044257~cellular protein catabolic process	147	5,617119	2,94E-06	UBXN2A, L
GOTERM_BP_ALL	GO:0044248~cellular catabolic process	334	12,76271	3,02E-06	SCOC, RNH
GOTERM_BP_ALL	GO:0045893~positive regulation of transcription, DNA-templated	273	10,43179	3,02E-06	RB1, ATF1,
GOTERM_BP_ALL	GO:1903508~positive regulation of nucleic acid-templated transcription	273	10,43179	3,02E-06	RB1, ATF1,
GOTERM_BP_ALL	GO:0006725~cellular aromatic compound metabolic process	910	34,77264	3,31E-06	ATF1, ATF2
GOTERM_BP_ALL	GO:0019941~modification-dependent protein catabolic process	125	4,776462	3,48E-06	UBXN2A, L
GOTERM_BP_ALL	GO:0044265~cellular macromolecule catabolic process	182	6,954528	3,59E-06	RNH1, UBX
GOTERM_BP_ALL	GO:0044763~single-organism cellular process	1534	58,61674	4,04E-06	ATF1, ATF2
GOTERM_BP_ALL	GO:0042592~homeostatic process	301	11,50172	4,38E-06	RB1, SPI1,
GOTERM_BP_ALL	GO:0051056~regulation of small GTPase mediated signal transduction	67	2,560183	4,43E-06	ARHGAP9,
GOTERM_BP_ALL	GO:0051179~localization	896	34,23768	4,7E-06	ATF2, SPI1
GOTERM_BP_ALL	GO:0051234~establishment of localization	686	26,21322	4,88E-06	ATF2, IPO1
GOTERM_BP_ALL	GO:0043632~modification-dependent macromolecule catabolic process	126	4,814673	5E-06	UBXN2A, L
GOTERM_BP_ALL	GO:2000112~regulation of cellular macromolecule biosynthetic process	660	25,21972	5,09E-06	ATF1, RB1,
GOTERM_BP_ALL	GO:0006810~transport	663	25,33435	5,51E-06	ATF2, IPO1
GOTERM_BP_ALL	GO:0045944~positive regulation of transcription from RNA polymerase II promoter	208	7,948032	5,84E-06	RB1, ATF1,
GOTERM_BP_ALL	GO:1901360~organic cyclic compound metabolic process	936	35,76614	6,77E-06	ATF1, ATF2
GOTERM_BP_ALL	GO:0044260~cellular macromolecule metabolic process	1290	49,29308	7,16E-06	ATF1, ATF2
GOTERM_BP_ALL	GO:0007154~cell communication	905	34,58158	7,46E-06	ATF1, ATF2
GOTERM_BP_ALL	GO:0010256~endomembrane system organization	137	5,235002	7,87E-06	WASHC4, I
GOTERM_BP_ALL	GO:0044237~cellular metabolic process	1539	58,8078	8,01E-06	ATF1, ATF2
GOTERM_BP_ALL	GO:1902580~single-organism cellular localization	176	6,725258	8,52E-06	ATF2, IPO1

GOTERM_BP_ALL	GO:0009056~catabolic process	380	14,52044	8,63E-06	SCOC, PGL
GOTERM_BP_ALL	GO:0044249~cellular biosynthetic process	907	34,65801	8,75E-06	ATF1, ATF2
GOTERM_BP_ALL	GO:0044802~single-organism membrane organization	148	5,655331	9,23E-06	ATF2, ACA
GOTERM_BP_ALL	GO:0009894~regulation of catabolic process	155	5,922812	9,57E-06	SCOC, UBX
GOTERM_BP_ALL	GO:0044093~positive regulation of molecular function	262	10,01146	1,11E-05	EPHB6, RB
GOTERM_BP_ALL	GO:0097190~apoptotic signaling pathway	112	4,27971	1,15E-05	RB1, ATF2,
GOTERM_BP_ALL	GO:0033043~regulation of organelle organization	231	8,826901	1,47E-05	RB1, NCKA
GOTERM_BP_ALL	GO:0051052~regulation of DNA metabolic process	106	4,050439	1,56E-05	OTUD4, AT
GOTERM_BP_ALL	GO:0048523~negative regulation of cellular process	764	29,19373	1,64E-05	RB1, ATF2,
GOTERM_BP_ALL	GO:0006259~DNA metabolic process	180	6,878105	1,72E-05	OTUD4, AT
GOTERM_BP_ALL	GO:0048519~negative regulation of biological process	818	31,25716	1,75E-05	ATF2, ZNF
GOTERM_BP_ALL	GO:0060341~regulation of cellular localization	164	6,266718	2,09E-05	RB1, RIPOI
GOTERM_BP_ALL	GO:0006886~intracellular protein transport	176	6,725258	2,09E-05	ATF2, RIPC
GOTERM_BP_ALL	GO:0009059~macromolecule biosynthetic process	774	29,57585	2,16E-05	ATF1, ATF2
GOTERM_BP_ALL	GO:0001775~cell activation	191	7,298433	2,19E-05	EPHB6, AT
GOTERM_BP_ALL	GO:0023057~negative regulation of signaling	229	8,750478	2,22E-05	RB1, SPI1,
GOTERM_BP_ALL	GO:1902532~negative regulation of intracellular signal transduction	108	4,126863	2,46E-05	RIPOR1, SF
GOTERM_BP_ALL	GO:0031400~negative regulation of protein modification process	113	4,317921	2,53E-05	RB1, SPI1,
GOTERM_BP_ALL	GO:0010648~negative regulation of cell communication	228	8,712266	2,58E-05	RB1, SPI1,
GOTERM_BP_ALL	GO:0009987~cellular process	2175	83,11043	2,74E-05	ATF1, ATF2
GOTERM_BP_ALL	GO:1902589~single-organism organelle organization	288	11,00497	2,91E-05	RB1, NCKA
GOTERM_BP_ALL	GO:0010033~response to organic substance	464	17,73023	3,29E-05	ATF1, RB1,
GOTERM_BP_ALL	GO:0023052~signaling	890	34,00841	3,68E-05	ATF1, ATF2
GOTERM_BP_ALL	GO:0050863~regulation of T cell activation	74	2,827665	3,9E-05	EPHB6, SIF
GOTERM_BP_ALL	GO:0031329~regulation of cellular catabolic process	128	4,891097	3,95E-05	SCOC, EHM
GOTERM_BP_ALL	GO:0051348~negative regulation of transferase activity	62	2,369125	3,96E-05	RB1, GSK3
GOTERM_BP_ALL	GO:0010628~positive regulation of gene expression	333	12,72449	4,15E-05	RB1, ATF1,
GOTERM_BP_ALL	GO:0010498~proteasomal protein catabolic process	90	3,439052	4,17E-05	GSK3A, UB
GOTERM_BP_ALL	GO:0051247~positive regulation of protein metabolic process	275	10,50822	4,22E-05	ATF2, SPI1
GOTERM_BP_ALL	GO:0045321~leukocyte activation	169	6,457776	4,26E-05	EPHB6, AT
GOTERM_BP_ALL	GO:0042325~regulation of phosphorylation	253	9,667558	4,56E-05	EPHB6, RB
GOTERM_BP_ALL	GO:0007032~endosome organization	26	0,993504	4,6E-05	WASHC4, I
GOTERM_BP_ALL	GO:0016070~RNA metabolic process	721	27,55063	4,63E-05	ATF1, RB1,

GOTERM_BP_ALL	GO:0030705~cytoskeleton-dependent intracellular transport	46	1,757738	4,73E-05	CCDC186,
GOTERM_BP_ALL	GO:0044839~cell cycle G2/M phase transition	38	1,452044	4,8E-05	BRSK1, BL
GOTERM_BP_ALL	GO:0007265~Ras protein signal transduction	70	2,674818	4,8E-05	RB1, ITGB
GOTERM_BP_ALL	GO:0044700~single organism signaling	883	33,74092	4,9E-05	ATF1, ATF
GOTERM_BP_ALL	GO:0042110~T cell activation	99	3,782958	5E-05	EPHB6, AT
GOTERM_BP_ALL	GO:0007165~signal transduction	825	31,52465	5E-05	ATF1, ATF
GOTERM_BP_ALL	GO:0000086~G2/M transition of mitotic cell cycle	35	1,337409	5,19E-05	BRSK1, BL
GOTERM_BP_ALL	GO:0043433~negative regulation of sequence-specific DNA binding transcription factor activity	42	1,604891	5,6E-05	PSMD10,
GOTERM_BP_ALL	GO:0010243~response to organonitrogen compound	159	6,075659	5,88E-05	RB1, ATF1,
GOTERM_BP_ALL	GO:0006469~negative regulation of protein kinase activity	50	1,910585	6,16E-05	RB1, DUSP
GOTERM_BP_ALL	GO:0009719~response to endogenous stimulus	268	10,24073	6,34E-05	RB1, ATF1,
GOTERM_BP_ALL	GO:0032386~regulation of intracellular transport	92	3,515476	6,69E-05	RIPOR1, G
GOTERM_BP_ALL	GO:0071901~negative regulation of protein serine/threonine kinase activity	34	1,299198	6,82E-05	RB1, CEBP
GOTERM_BP_ALL	GO:0051174~regulation of phosphorus metabolic process	277	10,58464	6,91E-05	EPHB6, RB
GOTERM_BP_ALL	GO:0044033~multi-organism metabolic process	32	1,222774	7,41E-05	IFITM3, GS
GOTERM_BP_ALL	GO:0019220~regulation of phosphate metabolic process	276	10,54643	7,61E-05	EPHB6, RB
GOTERM_BP_ALL	GO:1901698~response to nitrogen compound	179	6,839893	7,63E-05	RB1, ATF1,
GOTERM_BP_ALL	GO:0050868~negative regulation of T cell activation	33	1,260986	7,7E-05	FGL2, PTE
GOTERM_BP_ALL	GO:2001233~regulation of apoptotic signaling pathway	77	2,9423	7,7E-05	RB1, GSK3
GOTERM_BP_ALL	GO:0046649~lymphocyte activation	144	5,502484	8,18E-05	EPHB6, AT
GOTERM_BP_ALL	GO:0010035~response to inorganic substance	108	4,126863	8,94E-05	ATF1, ATF
GOTERM_BP_ALL	GO:0040007~growth	156	5,961024	9,83E-05	RB1, ATF2,

List Total	Pop Hits	Pop Total	Fold Enricl	Bonferron	Benjamini	FDR
2313	4330	19308	1,309012	1,12E-12	1,36E-12	1,31E-12
2313	4274	19308	1,306632	3,37E-12	1,36E-12	1,31E-12
2313	4052	19308	1,314356	5,62E-12	1,36E-12	1,31E-12
2313	4052	19308	1,314356	5,62E-12	1,36E-12	1,31E-12
2313	3271	19308	1,360217	6,75E-12	1,45E-12	1,39E-12
2313	3933	19308	1,311675	3,6E-11	5,68E-12	5,45E-12
2313	3687	19308	1,324477	3,94E-11	5,68E-12	5,45E-12
2313	4328	19308	1,290329	5,51E-11	6,82E-12	6,54E-12
2313	3873	19308	1,308286	1,25E-10	1,38E-11	1,33E-11
2313	3726	19308	1,315094	1,57E-10	1,57E-11	1,5E-11
2313	3708	19308	1,312473	3,06E-10	2,59E-11	2,48E-11
2313	3846	19308	1,304448	3,27E-10	2,59E-11	2,48E-11
2313	3711	19308	1,311412	3,36E-10	2,59E-11	2,48E-11
2313	4415	19308	1,276247	4,4E-10	3,14E-11	3,01E-11
2313	4408	19308	1,274486	6,55E-10	4,37E-11	4,19E-11
2313	4567	19308	1,261188	2,58E-09	1,61E-10	1,55E-10
2313	2549	19308	1,378713	3,31E-09	1,95E-10	1,87E-10
2313	2783	19308	1,352773	7,18E-09	3,99E-10	3,83E-10
2313	13478	19308	1,088817	1,16E-08	6,11E-10	5,86E-10
2313	3045	19308	1,329585	1,31E-08	6,55E-10	6,28E-10
2313	12039	19308	1,102474	5,61E-08	2,67E-09	2,56E-09
2313	12707	19308	1,094444	6,36E-08	2,89E-09	2,77E-09
2313	6682	19308	1,181806	1,45E-07	6,31E-09	6,05E-09
2313	6015	19308	1,196281	1,71E-07	7,12E-09	6,83E-09
2313	2784	19308	1,322303	4,77E-07	1,91E-08	1,83E-08
2313	3673	19308	1,26589	9,73E-07	3,74E-08	3,59E-08
2313	2663	19308	1,322827	1,47E-06	5,45E-08	5,23E-08
2313	7081	19308	1,163548	1,74E-06	6,22E-08	5,97E-08
2313	2083	19308	1,370561	2,03E-06	6,99E-08	6,7E-08
2313	1654	19308	1,423231	2,18E-06	7,28E-08	6,98E-08
2313	2247	19308	1,352259	2,35E-06	7,58E-08	7,27E-08

2313	3955	19308	1,247391	2,53E-06	7,89E-08	7,57E-08
2313	7582	19308	1,152722	2,75E-06	8,33E-08	7,99E-08
2313	2127	19308	1,361832	3,14E-06	9,23E-08	8,85E-08
2313	4409	19308	1,224971	5,72E-06	1,64E-07	1,57E-07
2313	1494	19308	1,435966	6,63E-06	1,84E-07	1,77E-07
2313	3084	19308	1,28029	9,34E-06	2,52E-07	2,42E-07
2313	6944	19308	1,158855	9,94E-06	2,62E-07	2,51E-07
2313	4217	19308	1,225318	1,63E-05	4,18E-07	4,01E-07
2313	3632	19308	1,248003	1,8E-05	4,4E-07	4,22E-07
2313	1424	19308	1,436209	1,81E-05	4,4E-07	4,22E-07
2313	1786	19308	1,378803	2,39E-05	5,69E-07	5,45E-07
2313	1403	19308	1,433907	2,8E-05	6,52E-07	6,25E-07
2313	1406	19308	1,430848	3,44E-05	7,82E-07	7,49E-07
2313	2386	19308	1,315464	3,62E-05	8,04E-07	7,71E-07
2313	1920	19308	1,352137	6,97E-05	1,52E-06	1,45E-06
2313	994	19308	1,511638	8,45E-05	1,77E-06	1,7E-06
2313	3217	19308	1,255903	8,51E-05	1,77E-06	1,7E-06
2313	1982	19308	1,335111	0,000179	3,65E-06	3,5E-06
2313	1968	19308	1,336125	0,000187	3,74E-06	3,58E-06
2313	2111	19308	1,320748	0,000223	4,37E-06	4,19E-06
2313	2298	19308	1,304086	0,000232	4,46E-06	4,28E-06
2313	7035	19308	1,142678	0,000259	4,8E-06	4,6E-06
2313	1016	19308	1,487122	0,000259	4,8E-06	4,6E-06
2313	1968	19308	1,331883	0,000274	4,99E-06	4,78E-06
2313	4292	19308	1,201961	0,00039	6,78E-06	6,5E-06
2313	1741	19308	1,35211	0,000393	6,78E-06	6,5E-06
2313	3623	19308	1,225759	0,000393	6,78E-06	6,5E-06
2313	3651	19308	1,223217	0,000477	8,09E-06	7,76E-06
2313	6962	19308	1,140271	0,000557	9,28E-06	8,9E-06
2313	1952	19308	1,321418	0,000815	1,34E-05	1,28E-05
2313	1034	19308	1,461234	0,000941	1,52E-05	1,46E-05
2313	3261	19308	1,233837	0,00098	1,56E-05	1,49E-05
2313	1078	19308	1,448053	0,001058	1,65E-05	1,59E-05

2313	3354	19308	1,227003	0,001389	2,12E-05	2,03E-05
2313	853	19308	1,50707	0,001398	2,12E-05	2,03E-05
2313	7829	19308	1,122752	0,001483	2,21E-05	2,12E-05
2313	5214	19308	1,167127	0,001878	2,76E-05	2,65E-05
2313	250	19308	2,003424	0,001964	2,85E-05	2,73E-05
2313	5081	19308	1,169748	0,002003	2,86E-05	2,75E-05
2313	1979	19308	1,307608	0,002094	2,95E-05	2,83E-05
2313	1900	19308	1,313649	0,002378	3,31E-05	3,17E-05
2313	7159	19308	1,129882	0,002559	3,51E-05	3,37E-05
2313	1844	19308	1,317327	0,002715	3,67E-05	3,52E-05
2313	1861	19308	1,314265	0,003088	4,07E-05	3,91E-05
2313	1597	19308	1,343352	0,003109	4,07E-05	3,91E-05
2313	1329	19308	1,381845	0,003131	4,07E-05	3,91E-05
2313	1804	19308	1,318773	0,003445	4,42E-05	4,24E-05
2313	290	19308	1,899799	0,003611	4,58E-05	4,39E-05
2313	792	19308	1,507206	0,004004	5,01E-05	4,81E-05
2313	2899	19308	1,238175	0,004338	5,37E-05	5,15E-05
2313	975	19308	1,446917	0,004996	6,11E-05	5,86E-05
2313	6535	19308	1,13558	0,005168	6,24E-05	5,99E-05
2313	2231	19308	1,275899	0,005567	6,65E-05	6,37E-05
2313	922	19308	1,457661	0,00595	7,02E-05	6,73E-05
2313	2046	19308	1,289268	0,00604	7,04E-05	6,75E-05
2313	881	19308	1,468647	0,006341	7,31E-05	7,01E-05
2313	4817	19308	1,168006	0,007089	8,08E-05	7,75E-05
2313	933	19308	1,449423	0,007948	8,97E-05	8,6E-05
2313	1678	19308	1,318304	0,00984	0,00011	0,000105
2313	770	19308	1,496063	0,009975	0,00011	0,000106
2313	923	19308	1,447038	0,010366	0,000113	0,000109
2313	5912	19308	1,142288	0,010511	0,000114	0,000109
2313	1331	19308	1,360954	0,011603	0,000124	0,000119
2313	1770	19308	1,306376	0,011719	0,000124	0,000119
2313	1038	19308	1,415393	0,011864	0,000124	0,000119
2313	6840	19308	1,126438	0,012259	0,000127	0,000122

2313	2236	19308	1,26558	0,012969	0,000133	0,000128
2313	6954	19308	1,123577	0,014448	0,000146	0,00014
2313	5017	19308	1,158049	0,014531	0,000146	0,00014
2313	6710	19308	1,127113	0,015416	0,000154	0,000148
2313	454	19308	1,654811	0,016015	0,000158	0,000152
2313	4272	19308	1,17437	0,019022	0,000186	0,000179
2313	450	19308	1,65097	0,020166	0,000196	0,000188
2313	2187	19308	1,2634	0,02076	0,0002	0,000192
2313	687	19308	1,506699	0,025614	0,000245	0,000235
2313	2672	19308	1,230896	0,026225	0,000248	0,000238
2313	841	19308	1,449167	0,029189	0,000273	0,000262
2313	848	19308	1,447049	0,029337	0,000273	0,000262
2313	2219	19308	1,256466	0,030125	0,000273	0,000262
2313	1761	19308	1,294091	0,030135	0,000273	0,000262
2313	1761	19308	1,294091	0,030135	0,000273	0,000262
2313	6770	19308	1,122056	0,032984	0,000297	0,000285
2313	698	19308	1,494914	0,034642	0,000309	0,000297
2313	1099	19308	1,382405	0,035742	0,000316	0,000303
2313	11989	19308	1,068081	0,040058	0,000352	0,000338
2313	1979	19308	1,269645	0,043428	0,000379	0,000364
2313	317	19308	1,764319	0,043869	0,00038	0,000365
2313	6672	19308	1,121021	0,046484	0,0004	0,000384
2313	4978	19308	1,150352	0,048206	0,000412	0,000395
2313	710	19308	1,481405	0,049357	0,000418	0,000401
2313	4773	19308	1,154288	0,050198	0,000422	0,000405
2313	4799	19308	1,153253	0,054318	0,000454	0,000435
2313	1296	19308	1,339738	0,05742	0,000477	0,000457
2313	7011	19308	1,114442	0,066217	0,000548	0,000526
2313	9942	19308	1,083123	0,069954	0,000576	0,000552
2313	6764	19308	1,11688	0,072724	0,000595	0,00057
2313	793	19308	1,442145	0,076592	0,000623	0,000597
2313	12058	19308	1,06543	0,077921	0,000629	0,000603
2313	1071	19308	1,371781	0,08263	0,000663	0,000636

2313	2598	19308	1,220973	0,083673	0,000667	0,00064
2313	6784	19308	1,116049	0,084801	0,000671	0,000644
2313	873	19308	1,415172	0,089219	0,000703	0,000674
2313	923	19308	1,401818	0,092386	0,000723	0,000694
2313	1708	19308	1,280487	0,105979	0,00083	0,000796
2313	625	19308	1,49589	0,110242	0,000859	0,000824
2313	1484	19308	1,299391	0,138031	0,001084	0,00104
2313	588	19308	1,50484	0,146221	0,001146	0,001098
2313	5648	19308	1,129173	0,15291	0,001194	0,001145
2313	1113	19308	1,350016	0,159485	0,001241	0,00119
2313	6089	19308	1,121422	0,162132	0,001255	0,001203
2313	1001	19308	1,367639	0,190464	0,001482	0,001421
2313	1088	19308	1,350347	0,190991	0,001482	0,001421
2313	5742	19308	1,125225	0,196476	0,001519	0,001457
2313	1198	19308	1,330878	0,198784	0,001528	0,001466
2313	1478	19308	1,29337	0,201218	0,001539	0,001476
2313	608	19308	1,482797	0,220881	0,001698	0,001628
2313	643	19308	1,466997	0,226304	0,001734	0,001662
2313	1473	19308	1,292093	0,229759	0,001752	0,00168
2313	17752	19308	1,02276	0,242474	0,001851	0,001775
2313	1927	19308	1,247592	0,255129	0,001951	0,00187
2313	3287	19308	1,178365	0,283715	0,002195	0,002105
2313	6704	19308	1,108199	0,311058	0,002435	0,002335
2313	384	19308	1,608652	0,326212	0,002564	0,002459
2313	755	19308	1,415222	0,329837	0,002574	0,002468
2313	306	19308	1,691344	0,330687	0,002574	0,002468
2313	2279	19308	1,219724	0,343348	0,002676	0,002566
2313	492	19308	1,527	0,344782	0,002676	0,002566
2313	1838	19308	1,248961	0,34783	0,002688	0,002578
2313	1050	19308	1,343566	0,350103	0,002693	0,002583
2313	1674	19308	1,261615	0,369859	0,002868	0,002751
2313	91	19308	2,385029	0,372294	0,002875	0,002756
2313	5345	19308	1,126028	0,374226	0,002876	0,002758

2313	207	19308	1,855022	0,380632	0,002921	0,002801
2313	159	19308	1,995024	0,384677	0,00293	0,002809
2313	360	19308	1,623145	0,385115	0,00293	0,002809
2313	6659	19308	1,106913	0,390911	0,002969	0,002847
2313	556	19308	1,486353	0,397046	0,002994	0,002871
2313	6186	19308	1,113283	0,397136	0,002994	0,002871
2313	142	19308	2,057507	0,408686	0,003091	0,002964
2313	184	19308	1,905431	0,432718	0,003315	0,003179
2313	984	19308	1,34885	0,448896	0,003464	0,003322
2313	234	19308	1,783675	0,46421	0,003607	0,003459
2313	1796	19308	1,245633	0,473703	0,003689	0,003537
2313	512	19308	1,499959	0,492346	0,003874	0,003715
2313	138	19308	2,056655	0,498773	0,003924	0,003763
2313	1866	19308	1,239167	0,503446	0,003955	0,003793
2313	127	19308	2,103332	0,527857	0,004216	0,004043
2313	1861	19308	1,238011	0,537403	0,004283	0,004107
2313	1136	19308	1,315335	0,538084	0,004283	0,004107
2313	133	19308	2,071209	0,541368	0,004283	0,004107
2313	412	19308	1,56011	0,541373	0,004283	0,004107
2313	882	19308	1,362874	0,563232	0,004526	0,00434
2313	627	19308	1,437864	0,595458	0,004918	0,004716
2313	972	19308	1,339738	0,630455	0,005381	0,00516
