# Supplementary material for: Replenished microglia partially rescue schizophrenia-related stress response
Source: Front Cell Neurosci. 2023 Sep 12;17:1254923. doi: 10.3389/fncel.2023.1254923 (PMC10522857; doi:10.3389/fncel.2023.1254923)
Supplement: Supplementary file 2 [file Data_Sheet_2.docx]

# Supplementary figures and figure legends

**Figure S1**


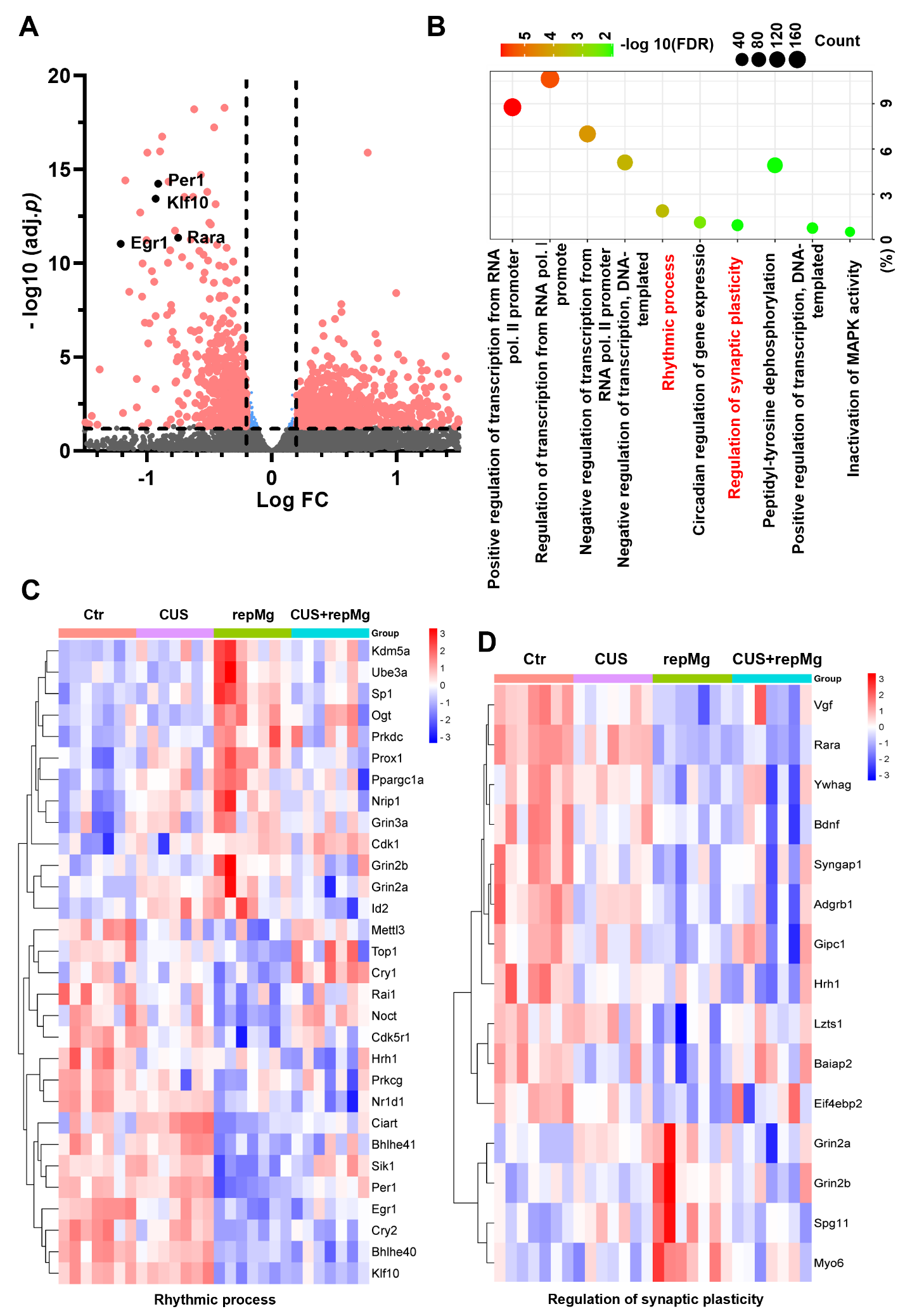


**Figure S1. DEGs in the mouse PFC and gene ontology (GO) analysis after regMg. (A)** Volcano plot showing expressions of DEGs compared between Ctr and repMg (*n*=7/group) from bulk RNA-seq analysis. Pink dots indicate significant DEGs. Those involved in synaptic plasticity and with values of -log10(FDR)>10 are highlighted in red dots. **(B)** GO-BP enrichment analysis of the DEGs showing top 10 pathways based on -log10(FDR) values of functional clustering and gene counts. **(C&D)** Heatmaps showing DEGs involved in rhythmic processes and synaptic plasticity among the 4 experimental groups.

**Figure S2**


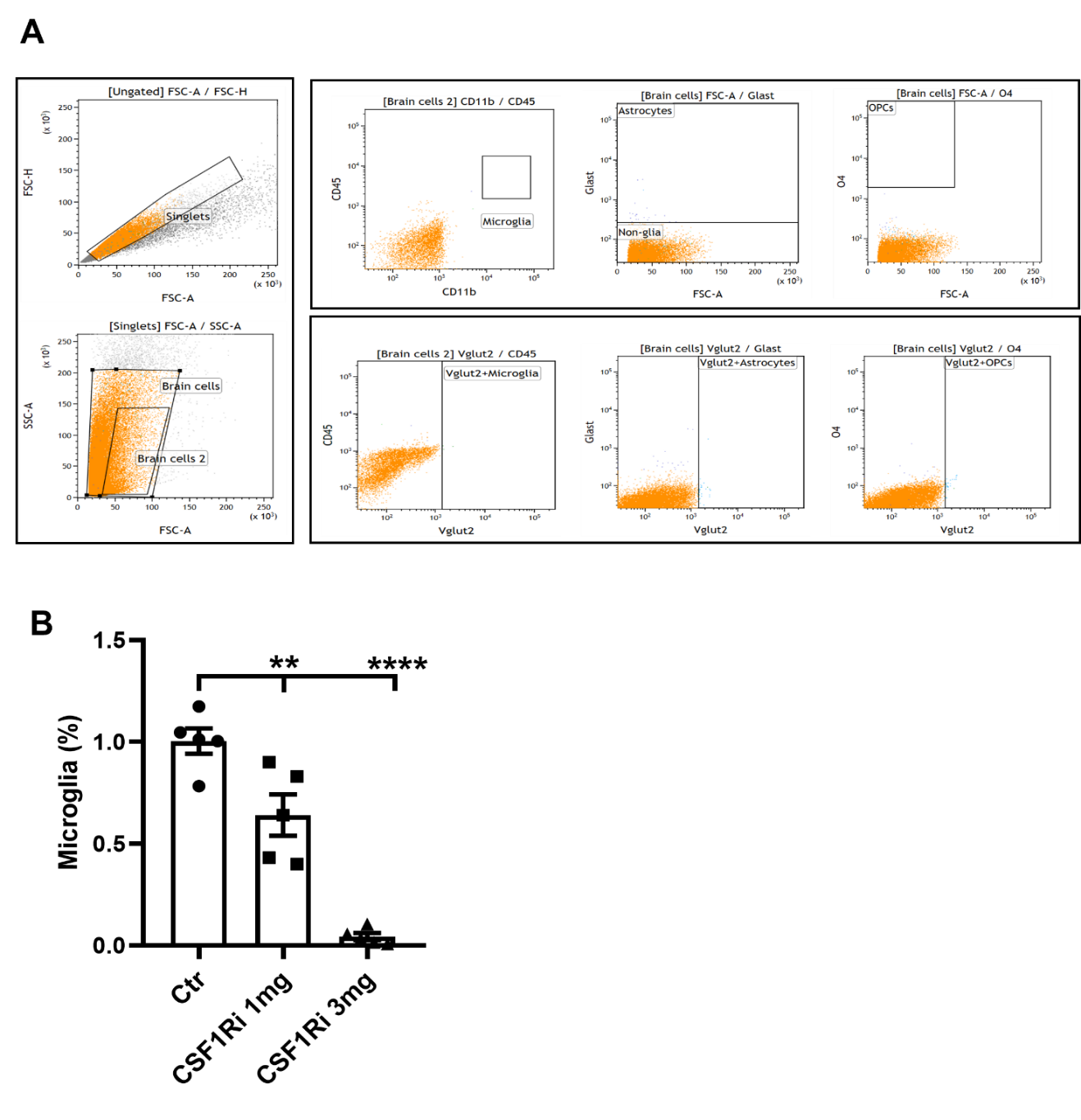


**Figure S2. Flow cytometry of mouse hippocampal glial cells.** (**A&B**) Dot plots represent gating strategy of flow cytometry markers for microglia, astrocytes, OPCs, and VGLUT2 (**A**) as well as their respective isotype controls (**B**). **(C)** CSF1Ri (PLX3397) dosage effect on microglial population after daily feeding in Nutella for 7 days, as quantified by flow cytometry immediately after drug withdrawal, showing 50% reduction by 1mg PLX3397/mouse/day and over 98% reduction by 3mg PLX3397/mouse/day.

**Figure S3**


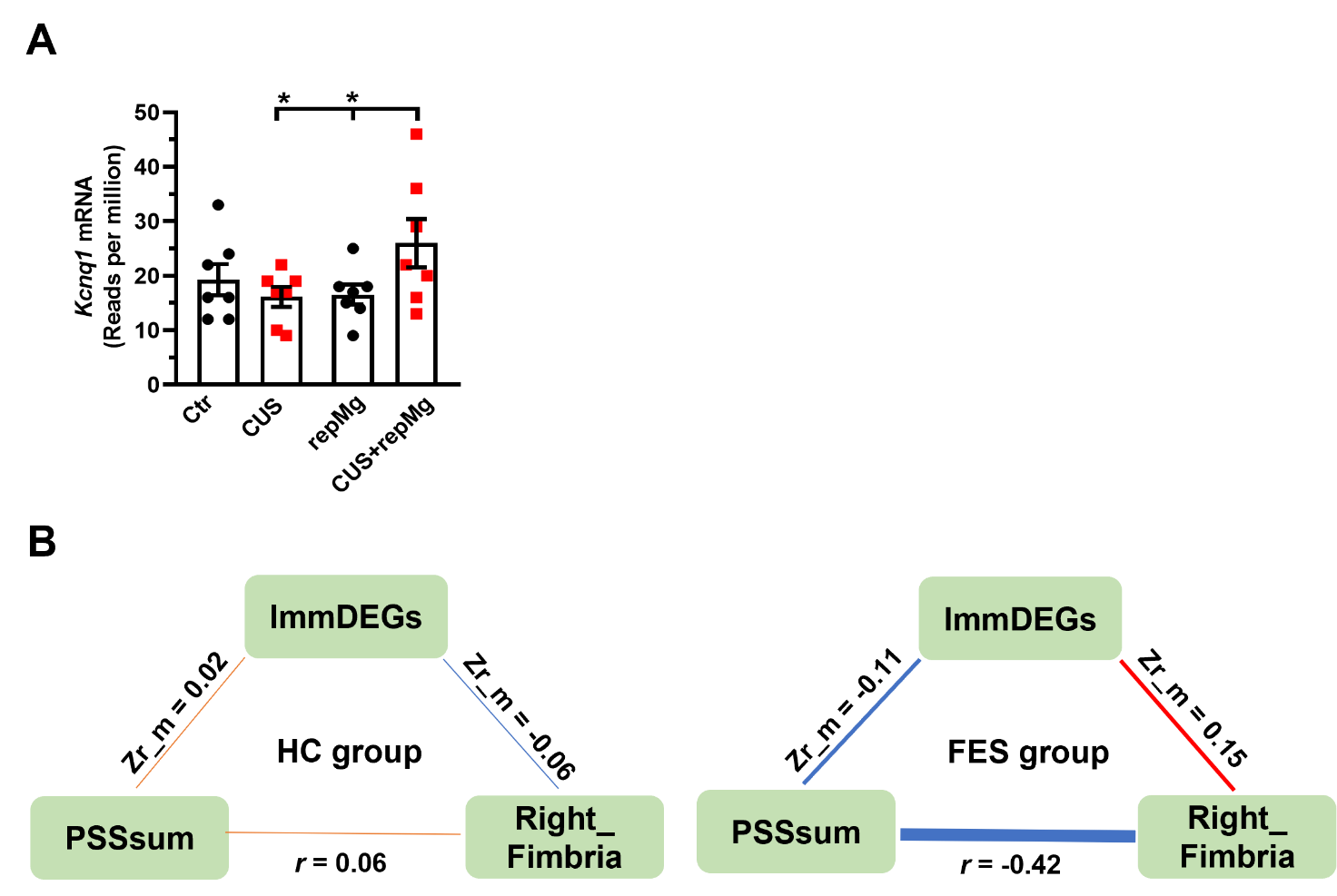


**Figure S3. *Kcnq1* expression in the PFC of CUS/repMg-exposed animals and correlation of blood immdev-DEGs with PSS and the right hippocampal fimbria in FES but not HC. (A)** mRNA levels of *Kcnq1* among the four groups of animals (Ctr, CUS, repMg, and CUS+repMg; *n*=7/group) as measured by bulk RNA-seq. * *p*<0.05 (* compared to CUS+repMg). Two-way ANOVA with LSD. **(B)** Diagrams summarizing inter-correlations among the 181 immdev-DEGs’ RNAseq counts, PSSsum scores, and right fimbria volumes in HC versus FES.
